# Cumulus expansion is impaired with advanced reproductive age due to loss of matrix integrity and reduced hyaluronan

**DOI:** 10.1111/acel.14004

**Published:** 2023-10-18

**Authors:** Elnur Babayev, Chanakarn Suebthawinkul, Dilan Gokyer, Wendena S. Parkes, Felipe Rivas, Mary Ellen Pavone, Adam R. Hall, Michele T. Pritchard, Francesca E. Duncan

**Affiliations:** ^1^ Department of Obstetrics and Gynecology, Feinberg School of Medicine Northwestern University Chicago Illinois USA; ^2^ Department of Obstetrics and Gynecology, Faculty of Medicine Chulalongkorn University Bangkok Thailand; ^3^ Department of Pharmacology, Toxicology, & Therapeutics, Institute for Reproductive and Developmental Sciences University of Kansas Medical Center Kansas City Kansas USA; ^4^ Virginia Tech‐Wake Forest University School of Biomedical Engineering and Sciences Wake Forest School of Medicine Winston‐Salem North Carolina USA

**Keywords:** aging, ART, cumulus expansion, hyaluronic acid, IVF, ovary

## Abstract

Reproductive aging is associated with ovulatory defects. Age‐related ovarian fibrosis partially contributes to this phenotype as short‐term treatment with anti‐fibrotic compounds improves ovulation in reproductively old mice. However, age‐dependent changes that are intrinsic to the follicle may also be relevant. In this study, we used a mouse model to demonstrate that reproductive aging is associated with impaired cumulus expansion which is accompanied by altered morphokinetic behavior of cumulus cells as assessed by time‐lapse microscopy. The extracellular matrix integrity of expanded cumulus–oocyte complexes is compromised with advanced age as evidenced by increased penetration of fluorescent nanoparticles in a particle exclusion assay and larger open spaces on scanning electron microscopy. Reduced hyaluronan (HA) levels, decreased expression of genes encoding HA‐associated proteins (e.g., *Ptx3* and *Tnfaip6*), and increased expression of inflammatory genes and matrix metalloproteinases underlie this loss of matrix integrity. Importantly, HA levels are decreased with age in follicular fluid of women, indicative of conserved reproductive aging mechanisms. These findings provide novel mechanistic insights into how defects in cumulus expansion contribute to age‐related infertility and may serve as a target to extend reproductive longevity.

## Graphical abstract

Reproductive aging is associated with impaired cumulus expansion. Cumulus–oocyte complexes from reproductively old mice demonstrate reduced hyaluronan (HA) levels and compromised extracellular matrix (ECM) integrity which is associated with dysregulated expression of ECM genes and increased transcript levels of inflammatory genes. Importantly, in humans, follicular fluid HA levels are decreased with age, indicative of conserved reproductive aging mechanisms.

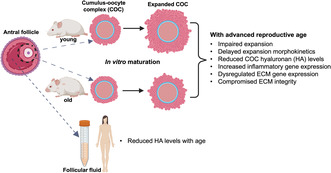

**Abbreviations**
ART: Assisted Reproductive Technologies
COC: cumulus–oocyte complex
ECM: extracellular matrix
GV: germinal vesicle
GVBD: germinal vesicle breakdown
HA: hyaluronan
IVF: in vitro fertilization
IVM: in vitro maturation
MII: metaphase of meiosis II
PMSG: pregnant mare serum gonadotropin

## 1 INTRODUCTION

The human ovary demonstrates functional decline in women beginning in their early 30s. Reproductive aging is characterized by a decrease in ovarian follicles which leads to decreased endocrine function and gonadal hormones and results in adverse health outcomes including heart disease, osteoporosis, and neurological dysfunction. In addition, there is a concordant decrease in oocyte quality which is associated with increased infertility and miscarriage rates (Broekmans et al., 2009). The shift to delayed childbearing in recent decades is associated with adverse fertility and maternal‐neonatal outcomes including chromosomal abnormalities, birth defects, increased risk of morbidity and mortality, high risk of cesarean delivery, preeclampsia, and postpartum hemorrhage (Aoyama et al., 2019; Hook, 1981; Li et al., 2022; Sauer, 2015; Sheen et al., 2018). Therefore, understanding the mechanisms of female reproductive aging is key to establishing new strategies to extend ovarian reproductive and endocrinological longevity and to improve infertility treatment outcomes.

Several gamete intrinsic changes occur with advanced reproductive age which underlie decreased egg quality, including an increase in chromosome and mitochondria abnormalities (Beverley et al., 2021; Duncan et al., 2012; Gruhn et al., 2019; Liu & Gao, 2022; Wang et al., 2011). However, oocytes do not develop and function in isolation but rather in a heterogenous ovarian microenvironment composed of granulosa, stromal, immune, and endothelial cells, and extracellular matrix (ECM) components (Fan et al., 2019; Wagner et al., 2020). The ovarian environment changes with age and is characterized by increased inflammation, oxidative damage, ovarian fibrosis, and increased stiffness (Amargant et al., 2020; Machlin et al., 2021; Wang et al., 2021). The levels of fibroinflammatory cytokines increase with reproductive aging and are associated with chronic inflammation and fibrosis in the ovarian stroma (Amargant et al., 2020; Machlin et al., 2021; Umehara et al., 2022). Coincidently, the levels of hyaluronan (HA), a glycosaminoglycan that consists of repeating disaccharide units and promotes tissue hydration and homeostasis, decrease in the ovarian stroma with advanced reproductive age (Amargant et al., 2020). In addition, there are significant changes in the immediate oocyte microenvironment which consists of cumulus cells and follicular fluid. Cumulus cells directly communicate with the oocyte via transzonal projections, regulate meiotic arrest, and support the metabolic needs of the oocyte during its maturation (Diaz et al., 2007; Dumesic et al., 2015; Rodgers & Irving‐Rodgers, 2010; Wigglesworth et al., 2013). Cumulus cells provide the oocyte with certain amino acids (l‐alanine), pyruvate and cholesterol to support its metabolic activity, since oocytes are unable to metabolize the glucose directly, synthesize cholesterol from acetate, or take up cholesterol via receptor‐mediated selective uptake (Su et al., 2009). Follicular fluid, which fills the antral follicle cavity, reflects the metabolism and synthetic capacity of the surrounding granulosa and cumulus cells (Babayev & Duncan, 2022; Rodgers & Irving‐Rodgers, 2010; Xie et al., 2023). With reproductive aging, both the cumulus cells and follicular fluid exhibit significant genomic, epigenetic, transcriptomic, proteomic, and metabolic alterations (Babayev & Duncan, 2022). Reproductive aging is also associated with ovulatory defects. After hormonal stimulation, reproductively old mice exhibit a greater number of oocytes trapped within unruptured luteinized follicles and expanded COCs which fail to ovulate compared to young mice (Mara et al., 2020). The incidence of anovulatory events also increases with advanced reproductive age in cows (Farin & Estill, 1993; Lopez‐Diaz & Bosu, 1992), mares (Carnevale, 1998; McCue & Squires, 2002), and humans (Wang et al., 2020). Ovarian fibrosis partly contributes to this ovulatory dysfunction because short‐term use of anti‐fibrotic compounds restores ovulation in reproductively old mice (Umehara et al., 2022). However, age‐related changes intrinsic to the follicle may also contribute to this phenotype.

In response to the Luteinizing Hormone (LH) surge at the mid‐menstrual cycle, the cumulus layer undergoes expansion which is necessary for optimal ovulation and successful fertilization; defects in cumulus expansion cause disruption in these processes and result in subfertility or infertility in mouse models (Davis et al., 1999; Fülöp et al., 2003; Hizaki et al., 1999; Salustri et al., 2004; Sato et al., 2001; Varani et al., 2002; Zhuo et al., 2001). Given the essential function of cumulus expansion in reproduction and increased ovulatory defects with advanced age, we tested the hypothesis that defective cumulus expansion may underlie age‐associated ovulatory dysregulation. We systematically analyzed cumulus expansion in a physiologic aging model using two different mouse strains. Our findings demonstrated an age‐dependent impairment of cumulus expansion due to compromised ECM integrity as a result of decreased HA levels and associated changes in the expression of inflammatory genes and ECM regulators. In addition, we analyzed HA polydispersity across age groups in follicular fluid of women undergoing infertility treatment and demonstrated that low‐molecular mass HA is the predominant form across the reproductive lifespan. However, overall follicular fluid HA levels decrease with age, indicating that similar reproductive aging mechanisms are likely conserved in humans. Our findings provide a novel mechanistic insight into how defective cumulus expansion contributes to age‐associated subfertility and may have implications for the treatment of infertility.

## 2 MATERIALS AND METHODS

### 2.1 Animals

All animal experiments described were approved by the Institutional Animal Care and Use Committee (Northwestern University) and performed in accordance with National Institutes of Health Guidelines. All CD1 female mice were obtained from Envigo. Reproductively young mice were 6–12 weeks of age. Reproductively middle‐age (8–10 months) and old (14–17 months) CD1 females were purchased as 7–8 months old, retired breeders and housed at our facility until appropriate age for experiments. Reproductively young (6–12 weeks) CB6F1 were purchased from Envigo. Reproductively old, retired breeder CB6F1 mice (14–17 months) were obtained from the Aged Rodent Colony at the National Institute on Aging. It is worth noting that the use of retired breeders is the standard for reproductive aging studies due to what is commercially available as well as the exceptional cost and time required to age virgin animals. Although no clear evidence shows that parity affects reproductive aging in mice, we cannot exclude that the observed phenotypic differences can at least partially be affected by prior breeding and births. Our experiments primarily utilized CB6F1 mice. Additional experiments were also performed with CD1 mice to assess conserved phenotypes and mechanisms across mouse strains. Based on a linear extrapolation of age, the 6–12‐week‐old mice are equivalent to women in their 20s, and 14–17 month old mice are equivalent to women in their 40s. This physiologic aging mouse model is well‐validated for the study of reproductive aging (Amargant et al., 2020; Duncan et al., 2017; Pan et al., 2008). Mice were housed in a controlled barrier facility at Northwestern University's Center for Comparative Medicine in Chicago under constant temperature, humidity, and light (14 h light/10 h dark). Upon arrival at Northwestern University, mice were fed a diet formulated to exclude soybean meal, Teklad Global 2916 chow (Envigo), and were provided food and water ad libitum.

### 2.2 COC collection, IVM, and expansion analysis

**FIGURE 1:**
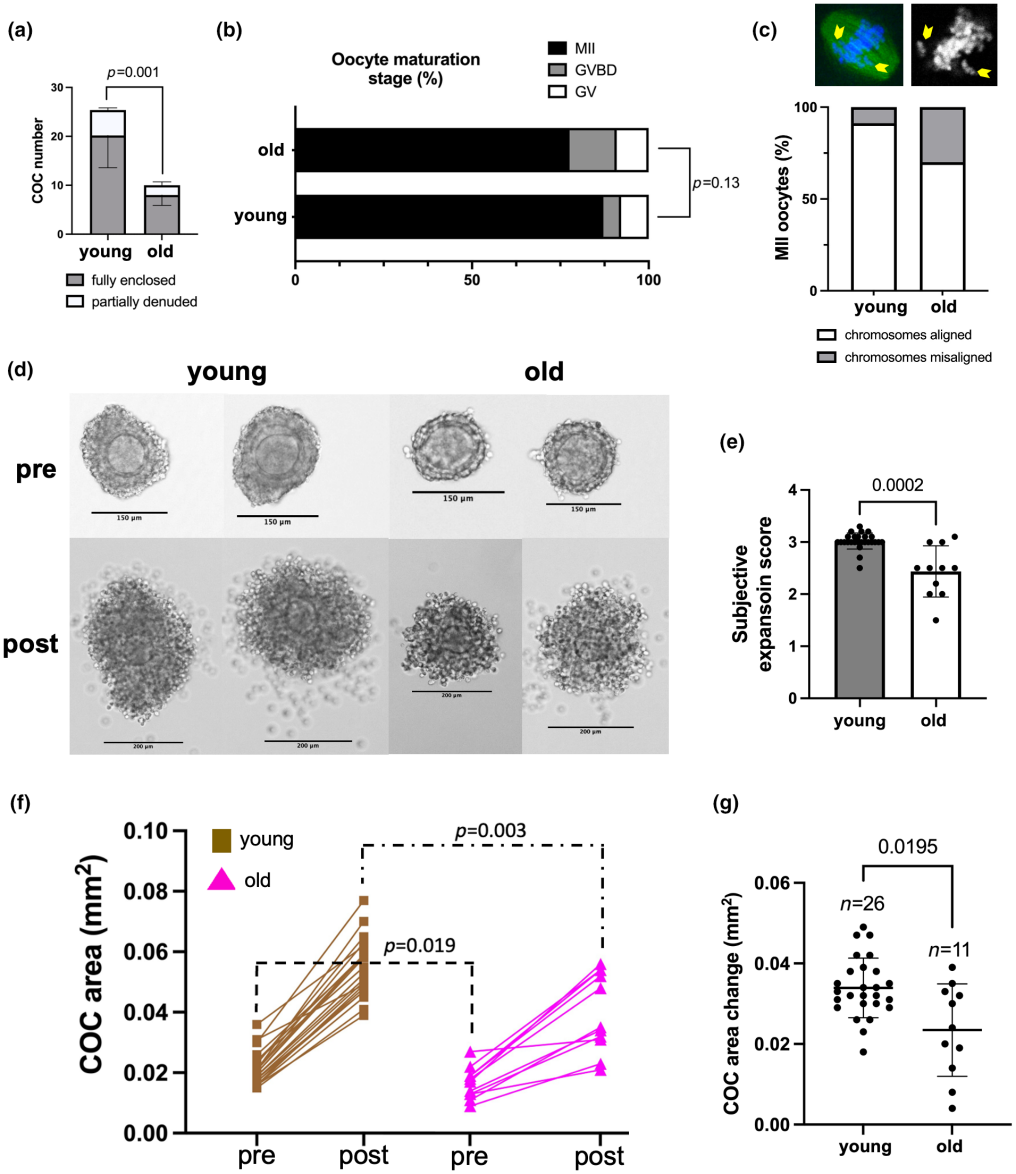
Cumulus expansion is impaired in COCs from reproductively old mice during IVM. (a) Significantly fewer COCs were obtained from reproductively old (14–17 months) mice after hormonal stimulation with PMSG. (b) Oocyte maturation stages were not significantly different between reproductively young and old mice after IVM of COCs in a conventional incubator. (c) Chromosome misalignment on metaphase II spindles showed a trend toward increased abnormalities with age (*p* = 0.15). (d) Representative images of COCs from reproductively young and old mice before and after cumulus expansion (scale bars: 150 μm for pre‐ and 200 μm for post‐expansion images) (e) COCs from reproductively old mice exhibited decreased subjective expansion scores. (f) COC area was decreased pre‐ and post‐expansion in COCs from reproductively old mice. (g) The change in COC area (post‐expansion area – pre‐expansion area) was significantly less in COCs from reproductively old mice during IVM (testing the difference between differences). Data are represented as mean ± SD. Experiments were repeated 5 times with the comparison of all COCs from one young and one old mouse per experiment (5–29 COCs per mouse). (b, c) Represent pooled analyses of oocytes from all experiments. Two‐sided Student's *t*‐test or Mann–Whitney *U* test was used to compare continuous variables depending on normality. Chi‐square test was used to compare categorical variables. *p* < 0.05 was considered statistically significant. GV, germinal vesicle; GVBD, germinal vesicle breakdown; IVM, *in vitro* maturation; MII, metaphase of meiosis II; PMSG, pregnant mare serum gonadotropin.

To maximize the yield of COCs, mice were hyperstimulated via intraperitoneal (IP) injections of 5 IU pregnant mare serum gonadotropin (PMSG) (ProSpec‐Tany TechnoGene, #HOR‐272), and ovaries were harvested 44–46 h post‐PMSG injection. Isolated ovaries were placed into dishes containing pre‐warmed Leibovitz's medium (L15) (Life Technologies Corporation) supplemented with 3 mg/mL polyvinylpyrrolidone (PVP) (Sigma‐Aldrich) and 0.5% (v/v) Penicillin–Streptomycin (PS) (Life Technologies Corporation) (L15/PVP/PS). Antral follicles were mechanically punctured with insulin syringes to release COCs from the ovaries. COCs were then transferred to L15/PVP/PS medium containing 2.5 μM milrinone (Sigma‐Aldrich), a PDE3A inhibitor that maintains oocytes arrested in prophase of meiosis I (Gilchrist et al., 2016). To induce synchronous meiotic maturation, milrinone was then removed through washes in L15/PVP/PS, and COCs were transferred to IVM media which supports cumulus expansion: α‐MEM/5%(v/v) Fetal bovine serum (FBS)/10 ng/mL Epidermal growth factor (EGF)/20 mM HEPES/0.25 mM pyruvate (purchased from Sigma‐Aldrich and Thermo Fisher Scientific; Suebthawinkul et al., 2022). COCs were cultured individually in 150 μL of media in wells of ultra‐low attachment 96‐well plates (Corning Costar, #CLS7007; Millipore Sigma). This experimental design allowed us to assess the expansion of individual COCs across age groups. This provides a more reliable assessment of cumulus expansion, given that mouse COCs form a clutch after ovulation, and post‐ovulatory assessment in both mice and humans is confounded by the incorporation of mural granulosa cells into the ovulated complex (Salustri et al., 1990, 1992). Cumulus expansion was analyzed using a subjective expansion scoring system as previously described (0 to +4, a score of 0 indicates no detectable response vs +4 indicates maximum degree of expansion, Figure S1c; Vanderhyden et al., 1990) and by measurements of pre‐ and post‐expansion COC area (Figure S1a) and average cumulus cell layer thickness (Figure S1b, measured at 3, 6, 9, and 12 o'clock positions and averaged) with ImageJ/Fiji (Schindelin et al., 2012, 2015). Since the pre‐expansion area and thickness were significantly different in COCs from reproductively young and old mice, we subtracted pre‐expansion measurements from post‐expansion measurements and compared these differences (i.e., difference between differences testing) (Figure 1g, Figures S2b, S4f,h, S5f,h).

### 2.3 Assessment of oocyte maturation status and spindle architecture and chromosome alignment in metaphase II‐arrested eggs

To accurately visualize the meiotic stage of the oocyte following IVM, surrounding cumulus cells were removed following a brief incubation in 0.25 mg/mL hyaluronidase (Sigma‐Aldrich). Oocytes that failed to mature and remained arrested at prophase of meiosis I were characterized by an intact nucleus or germinal vesicle (GV oocyte), whereas mature eggs arrested at metaphase of meiosis II (MII) were characterized by extrusion of the first polar body (PBI). Oocytes that had undergone germinal vesicle breakdown (GVBD) but lacked PBI were in between prophase I and MII and were referred to as a GVBD oocyte.

To assess the meiotic spindles and chromosome alignment during metaphase II, oocytes that had undergone polar body extrusion, immunocytochemistry was performed with a fluorescently conjugated antibody against alpha‐tubulin and a fluorescent DNA stain. Briefly, cells were fixed in 3.8% paraformaldehyde (PFA) (Electron Microscopy Sciences) with 0.1% TritonX‐100 (TX‐100; Sigma‐Aldrich) for 20 min at 37°C. After fixation, oocytes were washed with blocking buffer, 1× phosphate buffered saline (PBS) supplemented with 0.01% Tween‐20 (Sigma‐Aldrich), 0.02% Sodium azide (NaN3) (Sigma‐Aldrich), and 0.3% BSA (Sigma‐Aldrich), twice for 5 min. The cells were then incubated in permeabilization solution (1× PBS supplemented with 0.1% TX‐100, 0.02% NaN3, and 0.3% BSA) for 15 min at room temperature. The cells were washed again with blocking buffer and then incubated in alpha‐tubulin (11H10) Rabbit mAB 488 (1:100; Cell Signaling Technology #5063S) for 1 h at room temperature to visualize microtubules. The cells were then rinsed with blocking buffer three times for 20 min. The cells were then mounted on slides in Vectashield Antifade Mounting Medium with DAPI (4′,6‐diamidino‐2‐phenylindole; Vector Laboratories) to visualize chromosome alignment on meiotic spindles. Cells were imaged on a Leica SP5 inverted laser scanning confocal microscope (Leica Microsystems) using 405 nm and 488 nm lasers. The imaging was performed under 40× and 63× magnification, and Z‐stack thickness was 1 μm. Spindles and chromosome alignment were considered normal if spindles exhibited a bipolar structure with all chromosomes aligned on the metaphase plate. Only the oocytes with spindles parallelly oriented to the imaging plane were used for this analysis to accurately determine chromosome alignment (Balough et al., 2020). All images were processed and analyzed using FIJI (National Institutes of Health; Schindelin et al., 2012, 2015).

### 2.4 IVM of COCs within the EmbryoScope+™ and morphokinetic analysis of cumulus expansion

Time‐lapse experiments in EmbryoScope+™ were performed as previously described (Suebthawinkul et al., 2022, 2023). Briefly, EmbryoSlides (Vitrolife) were prepared the day before the experiment to allow media in the dishes to equilibrate in the EmbryoScope+™. The 16 microwells in the EmbryoSlides, each with a diameter of approximately 250 μm, allow reliable observation of cumulus expansion of one edge of the COC for ~12 h in most cases after which the cumulus cells expand beyond the well confines and imaging field of view. These microwells were filled according to the manufacturer's instructions with the specific maturation media designated for COCs as described above. The microwells were overlaid with 1.6 mL of mineral oil (Sigma‐Aldrich) and equilibrated in the EmbryoScope+™ for 9–24 h. COCs from reproductively young and old mice were loaded into the wells of the EmbryoSlide according to the manufacturer's instructions. EmbryoSlides were then loaded into the EmbryoScope+™. COCs were in vitro matured for a total of 16 h at 37°C in a humidified atmosphere of 5% CO_2_ in air. Images were taken every 10 min at 11 focal planes with low‐intensity red LED illumination with <0.5 s of light exposure per image. These conditions have minimal impact (if any) on gametes and are identical to those used for humans in ART. Following the COC expansion, oocyte maturation stage and chromosome alignment on metaphase II spindles were assessed as described above.

Morphokinetic parameters of cumulus layer expansion were evaluated with the EmbryoViewer software. The time point when COCs were loaded into the EmbryoScope+™ was set as the starting point. The distances of cumulus layer expansion were measured every 1 h at the same position as much as possible until the end of 16 h observation or until the cumulus layer expanded beyond the well limits which was 12 h for most COCs. The overall rate of cumulus layer expansion, the velocity of cumulus expansion at every 1 h, and the velocity of cumulus expansion at every 4 h were calculated by using this formula:

$$\begin{array}{c} \text{Velocity of expansion}\;\left(\mathrm{\mu m}/\min\right)\\ =\frac{\text{Distance}\;\mathrm{at}\;2\mathrm{nd}\;\text{time point}-\text{Distance}\;\mathrm{at}\;1\mathrm{st}\;\text{time point}}{\text{Time}\;\mathrm{at}\;2\mathrm{nd}\;\text{point}-\text{Time}\;\mathrm{at}\;1\mathrm{st}\;\text{point}} \end{array}$$

### 2.5 Detection and quantification of HA levels in expanded mouse COCs

To detect HA, we used an HA‐binding protein (HABP) assay (Suebthawinkul et al., 2022). In vitro matured COCs were fixed in 3.8% PFA for 30 min at room temperature. After fixation, COCs were washed in PBS twice for 5 min. Fixed COCs were then adhered to poly‐d‐lysine coated glass slides (100 μg/mL of poly‐d lysine in ddH_2_O) by pipetting COCs onto the slide and aspirating fluid from the periphery until COCs made contact with the slide. Endogenous avidin and biotin were blocked using an Avidin/Biotin blocking kit (Vector Laboratories). Avidin was applied for 15 min, slides were rinsed in PBS once, and then, biotin was applied for 15 min. Slides were rinsed in PBS a once again and were then incubated in normal goat serum (Vector Laboratories) for 20 min. Following a wash in PBS, biotinylated HABP (Calbiochem, #38599) diluted in normal goat serum (1:100) was added to all sections for 1‐h incubation at room temperature. Slides were then washed in PBSx1. Signal was amplified by incubating slides in ABC reagent (Vector Laboratories) for 30 min followed by TSA Plus Fluorescein System (Akoya Biosciences). Then, samples were mounted in Vectashield Antifade Mounting Medium with DAPI to stain cell nuclei. COCs were imaged on a Leica SP5 inverted laser scanning confocal microscope using 405 and 488 nm lasers. For HA analysis, the imaging was performed under 40x magnification, and the Z‐stack thickness was 1.5 μm. The imaging settings were kept constant for all samples. All images were processed and analyzed in a similar fashion using FIJI. HA content was measured as the mean intensity of signal per pixel of the image in selected regions of interest (ROI) per COC. To minimize the risk for subjective bias, nine ROIs per COC in at least three z‐planes were assessed to evaluate cellular and intercellular HA signal intensity. For cellular signal intensity assessment, each ROI comprised of 5 adjacent cumulus cells, and for intercellular signal intensity, same sized ROI was used in the intercellular area for both groups (Figure 3b insets).

### 2.6 Gene expression analysis of COCs using a customized RT^2^ Profiler PCR Hyaluronan Network Array

We generated a mouse Customized RT^2^ Profiler PCR HA Network Array 384G option (#CLAM34796E; Qiagen) to interrogate the differential expression of genes involved in the HA network in COCs obtained from reproductively young and old mice. The array profiles the expression of 88 genes broadly involved in HA biology within fibroinflammatory microenvironments (Figure S8). This assay was performed twice, and each experiment included pooled COCs from 5 reproductively young (78 COCs for the first and 94 COCs for the second experiment) and 5 old (28 COCs for the first and 36 COCs for the second experiment) mice. COCs were collected after 4 h of culture in maturation media. This is the timepoint when the expression of many genes involved in the cumulus expansion is up‐regulated. In addition, oocyte maturation stages should not be significantly different at this timepoint because polar body extrusion does not happen until at least 8‐ to 9‐h post‐IVM (Suebthawinkul et al., 2022), and even after overnight culture in a conventional incubator, there was no significant age‐dependent differences in oocyte maturation (Figure 1b). Total RNA was extracted using an RNeasy Micro Kit (#74004; Qiagen). cDNA was reverse transcribed using RT^2^ First Strand Kit (#330401; Qiagen) according to the manufacturer instructions. A 384 well‐plate QuantStudio5 (Applied Biosystems) machine was used for the PCR reaction. Five housekeeping genes (HKGs): *Gusb*, *Actb*, *Gapdh*, *B2m*, and *Hsp90ab1* were included in the array and used to normalize gene expression. Using the $2^{-\Delta \Delta C_{t}}$ method, fold changes (FC) in expression of each gene were calculated in COCs from reproductively old relative to reproductively young mice using the Qiagen GeneGlobe data analysis web tool (https://geneglobe.qiagen.com/us/analyze). The lower limit of detection was set at *C*_t_ = 35 as recommended by the array manufacturer to minimize over‐calculation of fold change for genes where the expression level is outside of acceptable assay sensitivity. The average fold changes of each experimental replicate were calculated. Up‐regulation and down‐regulation were defined as an average FC >+1.5 and −1.5, respectively. Log2 transformation of fold changes for all 88 genes were plotted to show expression changes relative to zero. In addition to COCs, we also examined the expression of genes involved in the HA network in whole ovaries and enriched stromal fractions (Amargant et al., 2020; Rowley, Amargant, et al., 2020) from ovaries of reproductively young (*N* = 4) and old (*N* = 4) mice using the customized RT^2^ Profiler PCR array as described.

### 2.7 PEA in mice expanded COCs

To determine the matrix integrity of expanded COCs, we adapted PEA to our system (Cohen et al., 2003; McLane et al., 2013; Simpson, 2019; Wei et al., 2019). COCs from reproductively young and old mice were matured in 50 mm glass‐bottom dishes (MatTek Corporation #P50G‐1.5‐14‐F). After IVM, well‐expanded COCs were fixed with 3.8% PFA for 30 min and stained with rhodamine phalloidin (1:50; Invitrogen #R415) for 1 h at room temperature to selectively stain actin filaments and NucBlue® Live Ready Probes® Reagent (2 drops/1 mL of media) to stain DNA. We incubated the stained COCs with fluorescent silica nanoparticles (excitation/emission: ~495/515 nm) of various sizes (100 and 500 nm, 20% v/v; Nanocs Inc.) for 1 h to visualize and evaluate the characteristics and porosity of the COC ECM. We then used 500‐nm particles for the comparative assessment of the ECM characteristics of expanded COCs from reproductively young and old mice. COCs were imaged on a Leica SP5 inverted laser scanning confocal microscope (Leica Microsystems) using 405 nm, 488 nm, and 543 nm lasers. The imaging was performed under 10× magnification with an optical section thickness of 1.5 μm. Image processing and analysis were performed using FIJI. To quantify ECM permeability to fluorescent nanoparticles, two methods were used (1) nanoparticle mean fluorescent intensity per pixel of the COC image selected as ROI (calculated as the total nanoparticle fluorescent intensity per COC/total number of pixels per COC image) and (2) % porosity which was calculated by dividing the area infiltrated by nanoparticles within the COC to the whole area occupied by the COC.

### 2.8 Scanning electron microscopy

Following IVM, COCs were placed on poly‐d‐lysine coated silicon wafers (Ted Pella #16010) and fixed with 2.5% EM grade glutaraldehyde (EMS #16200) and 2% paraformaldehyde (EMS #15711) in a 0.1M sodium cacodylate buffer solution pH 7.2 (EMS #11654), post‐fixed with 1% osmium tetroxide (#19150), and dehydrated with a graded series of ethanol prior to critical point drying in Tousimis Samdri‐795. Special attention was paid under a stereo microscope to ensure that the COCs were not dislodged from the wafer during each exchange of reagents. Wafers were placed upright within small porous capsules containing 80 μm pores and taken through a 15–20‐min purge cycle during the critical point drying process to ensure a slow and complete exchange from 200 proof ethanol to liquid CO_2_. Sample wafers were mounted to aluminum SEM stubs using silver paint (EMS #12630). A small line of the silver paint was carefully drawn up near the COCs from the edge of the stub under observation of a stereo microscope before sputter coating the samples with 7 nm of gold paladium in a Denton Desk IV sputtering system. Image data were gathered on a Hitachi S‐4800‐II cFEG SEM at 10 kV and an approximate working distance of 20 mm to optimize depth of field vs. resolution.

### 2.9 Isolation and measurement of HA levels in follicular fluid of women undergoing infertility treatment

Follicular fluid samples were obtained through Northwestern University IRB‐approved tissue repository, Reproductive Tissue Library (NU‐RTL, STU00072811). These samples were collected from patients undergoing infertility treatment via IVF (see Table S1 for patient characteristics). At the time of egg retrieval, follicular fluid from two dominant 18–20 mm follicles per participant was collected from these patients, samples were transferred to the research laboratory within 1 h of collection on ice, centrifuged at 300 *g* for 20 min to pellet cellular constituents, supernatant was transferred to sterile epitubes, snap frozen and stored at ‐80°C until HA isolation. HA was isolated following protocols reported previously (Amargant et al., 2020) with minor modifications. First, proteins were digested in each sample using a broad‐spectrum protease (proteinase K, #AM2548; Invitrogen) following the manufacturer's instructions. An equal volume of phenol:chloroform:isoamyl alcohol, 25:24:1 (#327111000; Thermo Fisher Scientific) was then added, mixed, and transferred to a phase‐lock gel tube (#2302830; QuantaBio) and centrifuged (14,000 *g* for 15 min at 20°C) to partition HA, nucleic acids, and other polysaccharides into an upper aqueous phase and proteins and other debris into a lower organic phase. The process was repeated twice with pure chloroform (#AC423555000; Thermo Fisher Scientific) to remove residual phenol. HA was quantified in the resulting solutions using an HA ELISA kit (Echelon; Product Number: K‐1200) following the manufacturer's instructions. All measurements were performed in triplicate.

To perform molecular weight fractionation and measure <50 kDa HA levels, HA was isolated as described above. Then, the aqueous layer containing HA was removed and transferred into the Vivaspin 500, 50,000 MW (molecular weight) cutoff column (Sartorius). The MW cutoff columns were centrifuged at 20°C for 10 min at 12,000 *g* which separated the sample into <50 and >50 kDa fractions. <50 kDa fraction of the follicular fluid was further diluted from the initial dilution of 1:2 by doing serial dilutions up to 1:32 using the RD5‐18 buffer. HA in the follicular fluid samples was measured using the Hyaluronan Quantikine ELISA Kit (R&D Systems, Catalog #DHYAL0) according to the manufacturer's instructions. R&D Systems kit was used for this experiment due to its assay range and sensitivity which is more appropriate for the <50 kDa fraction of human follicular fluid. The data were analyzed using a four‐parameter logistic curve (https://myassays.com/four‐parameter‐logistic‐curve.assay, accessed on November 1, 2022).

### 2.10 HA polydispersity analysis via solid‐state nanopore technology

After HA isolation from follicular fluid, HA molecules were captured selectively from each solvent‐extracted aqueous sample using biomagnetic precipitation. To form capture beads, Dynabeads™ M‐280 streptavidin‐coated superparamagnetic beads (#11206D; Thermo Fisher Scientific) were washed according to the manufacturer's directions and then incubated under gentle agitation for 1 h at room temperature in 1× PBS with biotinylated versican G1 domain (bVG1, #G‐HA02; Echelon Biosciences) at a ratio of 1 μg bVG1 per 100 μg beads. After thorough washing and resuspension in 1× PBS to a concentration of 10 mg/mL, bVG1‐conjugated beads were divided in 150 μL aliquots. Each aqueous sample was added to a bead aliquot (dropwise to avoid size bias in the capture process), and the entire mixture was incubated at room temperature for at least 2 h. Beads were then removed magnetically from solution and washed to remove non‐specific material. Finally, the beads were resuspended in high salt buffer (6M LiCl, 10 mM Tris, 1 mM EDTA, pH 8.0) for 1 h to elute HA before being pulled out of solution by magnetic field. The supernatant was collected for solid‐state nanopore (SSNP) analysis.

SSNPs consisting of a single pore (diameter 6–11 nm) in a 20‐nm thick silicon nitride membrane were either fabricated with an established Helium ion milling method (Yang et al., 2011) or produced commercially by conventional silicon processing techniques (Norcada). For an HA measurement (Rivas et al., 2018), a chip was rinsed with ethanol and water, dried under filtered air flow, treated with air plasma (30 W; Harrick Plasma) for 2 min per side, and placed into a custom 3D printed flow cell (Carbon). Measurement buffer (Rivas et al., 2022) (6M LiCl, 10 mM Tris, 1 mM EDTA, pH 8.0) was then introduced on both sides of the SSNP chip, and silver/silver chloride electrodes were used connect each reservoir to an Axopatch 200B patch‐clamp amplifier (Molecular Devices). Isolated HA—already suspended in measurement buffer from the elution process—was then introduced to one side of the SSNP, and measurements were performed by applying 200–300 mV and monitoring trans‐membrane current at a rate of 200 kHz using a 100 kHz four‐pole Bessel filter. Data were collected with a custom LabVIEW program (National Instruments), and analysis was performed with an additional 5 kHz low‐pass filter applied. Molecular translocations were marked by temporary reductions in the ionic current (events) and analyzed using an ionic current threshold of 5*σ* compared to baseline noise and including durations in the range of 25 μs – 2.5 ms. The area of each event was mapped to a molecular weight using a calibration curve (Rivas et al., 2018) produced with a comparable SSNP. Each data set consisted of a minimum of 500 individual events.

### 2.11 Statistics

Data are presented as the mean ± SD (or SEM), and each experiment was repeated at least three times. All results were graphed using GraphPad Prism Software Version 9.0.1. The normal distribution of the data was evaluated with the Shapiro–Wilk and Kolmogorov–Smirnov test. Analysis between groups of continuous variables was performed with two‐sided Student's *t*‐test or Mann–Whitney *U* test depending on distribution. Nested *t*‐test was used to compare the means between two age groups while accounting for the variables derived from the same mouse (i.e., COCs from the same mouse are not completely independent of each other and nested *t*‐test accounts for this). One‐way ANOVA test was used to analyze the differences between >2 normally distributed continuous variables followed by Tukey's multiple‐comparison tests. Categorical variables were analyzed with Fisher's exact test or chi‐square test. HA polydispersity was analyzed under a Box‐Cox transformation Ynew = (*Y* ^−0.4242^ – 1)/(−0.4242) using a nested *t*‐test to account for the correlation between measures on the same patient. *p* values < 0.05 were considered statistically significant. For experiments where numerous COCs were measured from multiple biological replicates that showed consistent results, we present findings of one experiment to avoid pooling results from multiple independent experiments or averaging the results from replicates to prevent artificially increasing or decreasing the statistical power, respectively.

## 3 RESULTS

### 3.1 Cumulus expansion is impaired with advanced reproductive age

To examine whether age‐dependent changes occur in cumulus expansion, we collected COCs from reproductively young (6–12 weeks) and old (14–17 months) CB6F1 mice and performed in vitro maturation (IVM) which allowed us to track this process in a controlled manner. As expected, given the age‐associated decline in follicle number, we collected significantly fewer COCs from reproductively old mice compared to young controls (10 ± 2 COCs/mouse vs. 25 ± 7 COCs/mouse, *p* = 0.001; Figure 1a). Following IVM, we did not see any differences in meiotic competence as most oocytes in both age cohorts reached the mature metaphase II‐arrested (MII) stage (Figure 1b). However, there was a trend toward reduced incidence of MII‐arrested eggs in reproductively old mice relative to young controls (77.6 ± 4.7% vs. 87.2 ± 2.6%, *p* = 0.13; Figure 1b). MII‐arrested eggs from reproductively old mice also exhibited a trend toward higher chromosome misalignment on the meiotic spindle which is consistent with the well‐documented increase in egg aneuploidy with age (30% vs. 8.7%, *p* = 0.15; Figure 1c) (Duncan et al., 2009; Merriman et al., 2012). We then evaluated cumulus expansion. Prior to IVM, COCs from reproductively old CB6F1 mice were smaller relative to young controls based on COC area (0.017 ± 0.005 mm^2^ vs. 0.021 ± 0.005 mm^2^, *p* < 0.05) and average cumulus cell layer thickness (21 ± 6 μm vs. 31 ± 9 μm, *p* < 0.05; Figure 1d,f; Figures S1 and S2a). In contrast, there was no difference in oocyte diameter within these COCs (Figure S3).

During IVM, the cumulus layer undergoes a dramatic expansion through production of a prominent ECM (Figure 1d). However, the degree of cumulus expansion was significantly compromised with age based on several quantitative parameters. Relative to young counterparts, COCs from reproductively old mice had a lower subjective expansion score (2.4 ± 0.5 vs. 3 ± 0.16, *p* < 0.01; Figure 1e), smaller post‐expansion COC area (0.04 ± 0.01 mm^2^ vs. 0.06 ± 0.01 mm^2^, *p* < 0.01; Figure 1f), and smaller post‐expansion average cumulus cell layer thickness (63 ± 23 μm vs. 90 ± 11 μm, *p* < 0.001; Figure S2a). This translated into a decreased overall change in old mice COC area (post‐expansion – pre‐expansion: 0.02 ± 0.01 mm^2^ vs. 0.03 ± 0.007, *p* < 0.05; Figure 1g) and average cumulus cell layer thickness (post‐expansion – pre‐expansion: 42 ± 21 μm vs. 59 ± 8 μm, *p* < 0.01) relative to young controls (Figure S2b). This impaired cumulus expansion with age was conserved across mouse strains as similar results were obtained in CD1 mice at 14–17 months of age (Figure S4). In this strain, we also evaluated a mid‐reproductive age time point (8–10 months) and did not observe differences in cumulus expansion at this intermediate age (Figure S5).

### 3.2 Cumulus expansion morphokinetics are altered with age

**FIGURE 2:**
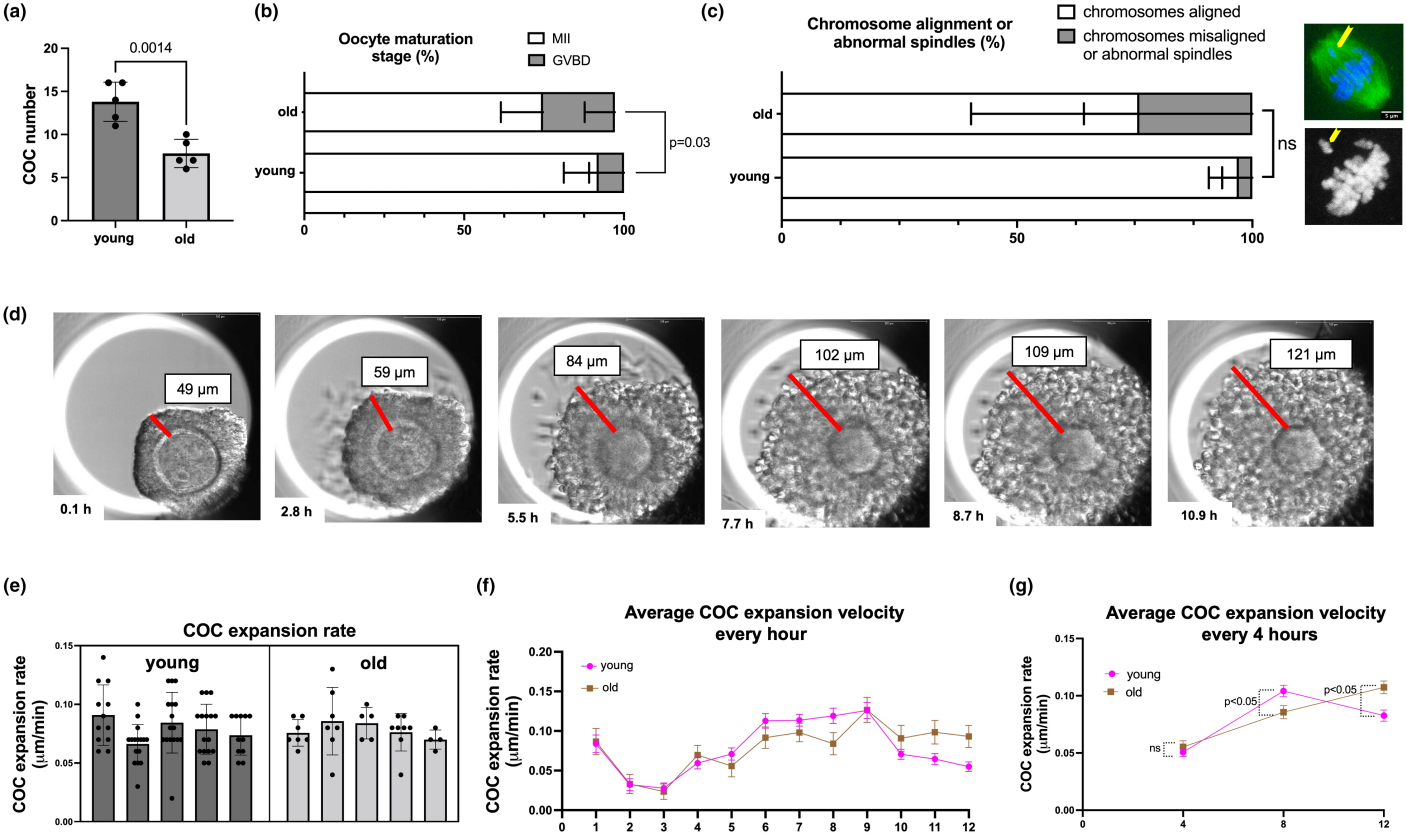
Cumulus expansion morphokinetics are altered in COCs from reproductively old mice during IVM in a closed time‐lapse incubator (EmbryoScope+™). (a) Significantly less COCs were obtained from reproductively old (14–17 months) mice after hormonal stimulation. (b) COCs from reproductively old mice showed reduced maturation rates after IVM. (c) Chromosome alignment on metaphase II spindles showed a trend toward increased abnormalities in older mice. (d) A representative series of montage images show cumulus layer expansion of an individual COC within EmbryoScope+™. (e) The average velocity of cumulus expansion did not differ with age. Each bar represents data from an individual mouse with individual dots in the scatter plot representing the expansion rate per COC. (f) COC expansion velocity change in 1‐h increments. (g) Morphokinetics (COC expansion velocity in 4‐h increments) of cumulus expansion differed with age. Experiments were repeated 5 times with the comparison of all COCs from one young and one old mouse per experiment (4–16 COCs per mouse). Data are represented as mean ± SD (except for 1G which is shown as mean ± SEM). Two‐sided Student's *t*‐test or Mann–Whitney *U* test was used to compare continuous variables depending on normality. Chi‐square test was used to compare categorical variables. Nested *t*‐test was used to compare average cumulus expansion velocity to account for the COCs from the same mouse. *p* < 0.05 was considered statistically significant. GVBD, germinal vesicle breakdown; IVM, *in vitro* maturation; MII, metaphase of meiosis II.

To further examine the dynamics of cumulus expansion, we performed IVM of COCs from reproductively young and old mice in a closed time‐lapse incubator system, EmbryoScope+™ (Suebthawinkul et al., 2022, 2023). Consistent with what we observed in our conventional IVM experiment, we collected fewer COCs from reproductively old mice compared to young controls (8 ± 2 COCs/mouse vs. 14 ± 2 COCs/mouse, *p* = 0.001; Figure 2a). Mice from reproductively old mice exhibited a reduced incidence of MII‐arrested eggs following IVM in this system (74.7 ± 13.2% vs. 92.1 ± 10.9%, *p* < 0.05) as well as a trend toward increased chromosomal misalignment on the meiotic spindle (24% vs 3%, *p* = 0.23; Figure 2b,c).

We assessed the overall rate of cumulus expansion during IVM, the average velocity of cumulus expansion at every 1 h, and the average velocity of cumulus expansion at every 4 h (Figure 2d–g, Video S1). The overall cumulus expansion rate was ~0.078 μm/min, and this was not different between age groups (Figure 2e). However, the expansion kinetics were different with age (Figure 2f,g). COCs from reproductively young mice expanded faster early (5–8 h) in culture (0.10 ± 0.04 μm/min vs. 0.085 ± 0.03 μm/min, *p* < 0.05) and then slowed down. In contrast, COCs from reproductively old mice took longer to reach this peak expansion velocity (9–12 h; 0.11 ± 0.03 μm/min vs. 0.08 ± 0.04 μm/min, *p* < 0.05), suggestive of a delayed morphokinetic profile of cumulus expansion (Figure 2f,g).

### 3.3 HA levels are reduced in COCs with age

**FIGURE 3:**
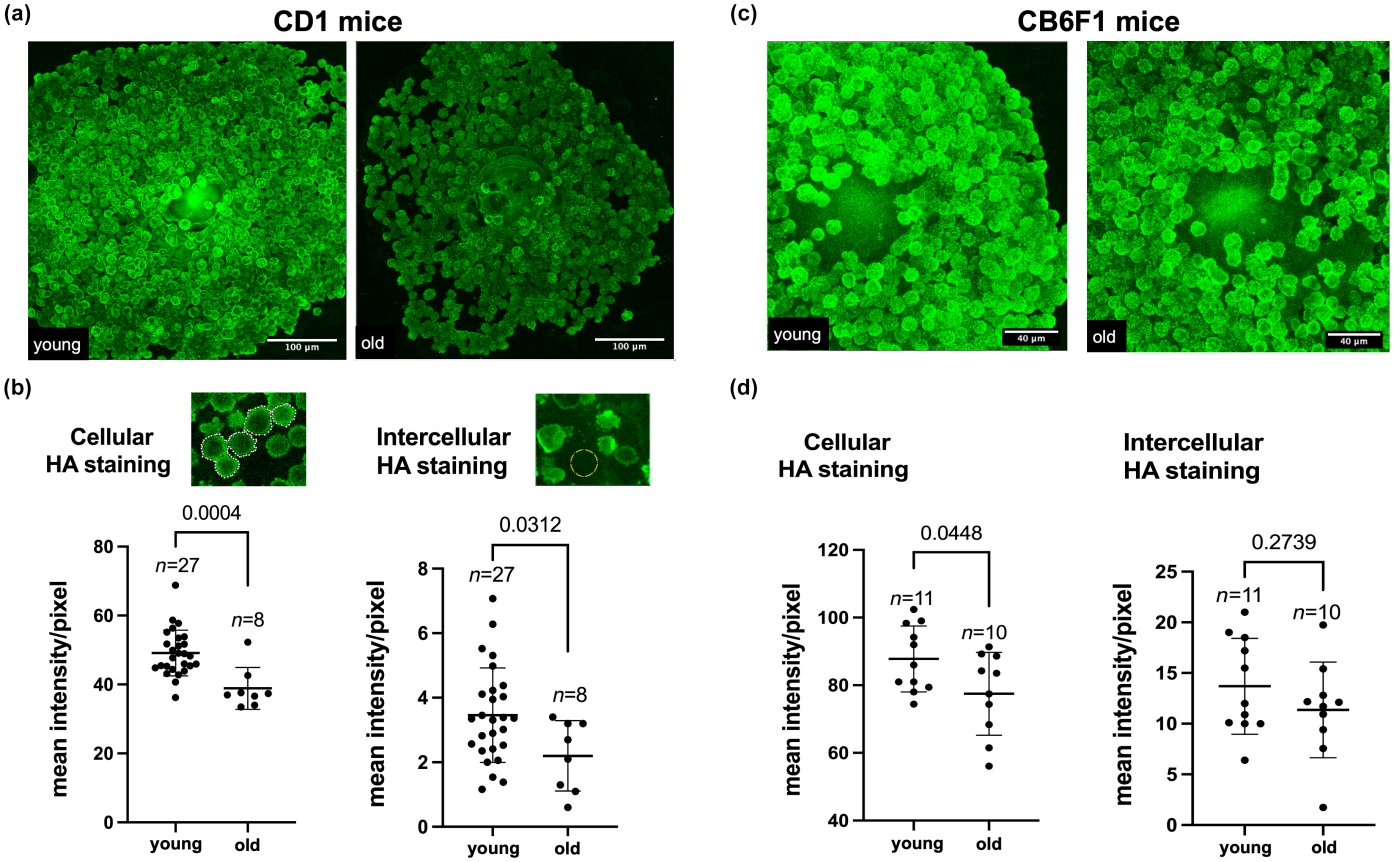
Hyaluronan (HA) levels are reduced in expanded COCs of reproductively old mice. (a, c) Representative images of expanded COCs from reproductively young (6–12 weeks) and old (14–17 months) CD1 (a) and CB6F1 (c) mice in which HA was visualized using the HABP assay (scale bars are 100 μm for (a) and 40 μm for (c). (b) Cellular and intercellular HA levels were decreased in expanded COCs from reproductively old CD1 mice. Experiments were repeated 3 times with the comparison of all COCs from one young and one old moue per experiment (8–27 COCs per mouse). (d) Cellular HA levels were decreased in expanded COCs from reproductively old CB6F1 mice and intercellular HA levels demonstrated a trend toward reduced levels. Experiments were repeated 5 times with the comparison of all COCs from one young and one old mouse per experiment (4–19 COCs per mouse). Data are represented as mean ± SD. Two‐sided Student's *t*‐test or Mann–Whitney *U* test was used to compare continuous variables depending on distribution. *p* < 0.05 was considered statistically significant.

To investigate the mechanism by which cumulus expansion may be impaired with age, we interrogated the HA matrix. Cumulus expansion is associated with and requires the deposition of large amounts of HA in the extracellular space which is primarily mediated by the activity of hyaluronan synthase 2 (Has2; Buccione et al., 1990; Eppig, 1979; Salustri et al., 1989, 2019). Therefore, we evaluated HA levels in the ECM of expanded COCs to assess whether reduced HA levels may be an underlying mechanism of compromised cumulus expansion in reproductively old mice. We detected HA in intact expanded COCs using an established hyaluronan‐binding protein (HABP assay; Amargant et al., 2020; Rowley, Rubenstein, et al., 2020; Suebthawinkul et al., 2022), and treatment with hyaluronidase abrogated the fluorescent signal and thereby validating specificity (Figure S6a). HA was detected both within the cumulus cells and in the intercellular matrix. HA is synthesized on the cytosolic side of the cell membrane by membrane‐embedded hyaluronan synthases and then released into the extracellular space (Hubbard et al., 2012). Stronger fluorescent intensity within cumulus cells compared to the intercellular space (Figure S6a–c) is likely due to higher HA concentration expected at the site of active synthesis. In some COCs, there was increased HA intensity in the cumulus cells closest to the oocyte (Figure S6b) and within blebs at the cumulus cell surface (Figure S6c). These findings are consistent with previous reports of increased HA levels in the corona radiata (Salustri et al., 2019; Scarchilli et al., 2007) and plasma membrane blebbing is associated with the migratory behavior of cumulus cells at the time of expansion (Akison et al., 2012; Dekel & Phillips, 1979; Fackler & Grosse, 2008; Kawashima et al., 2012; Relucenti et al., 2005; Salustri et al., 2019; Sutovsky et al., 1993). In contrast to the cumulus cells, HA levels were low in the oocyte but were detected at the oolemma, in the perivitelline space, and in the zona pellucida. This could represent a non‐specific staining or may suggest that HA is produced and secreted in part by the oocyte which would be consistent with *Has3* expression being enriched in the oocyte (Amargant et al., 2020) (Figure S6d). Importantly, HA levels were decreased in expanded COCs with age in two different strains of mice (CD1 and CB6F1) (Figure 3a–d). Expanded COCs from reproductively old CD1 mice had both lower cellular (mean fluorescence intensity/pixel: 38.9 ± 6.1 vs. 49.1 ± 6.6, *p* < 0.001) and intercellular (mean fluorescence intensity/pixel: 2.2 ± 1.1 vs. 3.5 ± 1.5, *p* < 0.05) HA levels compared to young counterparts (Figure 3a,b). In reproductively old CB6F1 mice, there was a reduction in cumulus cell associated HA (mean fluorescence intensity/pixel: 77.5 ± 12.2 vs. 87.8 ± 9.7, *p* < 0.05), and a trend toward reduced intercellular HA (mean fluorescence intensity/pixel: 11.4 ± 4.7 vs. 13.7 ± 4.7, *p* = 0.27; Figure 3c,d). No difference in HA levels was observed between expanded COCs of reproductively young and mid‐reproductive age CD1 mice, demonstrating that observed changes in HA are a phenomenon of advanced reproductive age (Figure S7a,b).

### 3.4 Alterations in HA‐related gene expression are associated with impaired cumulus expansion with age

**FIGURE 4:**
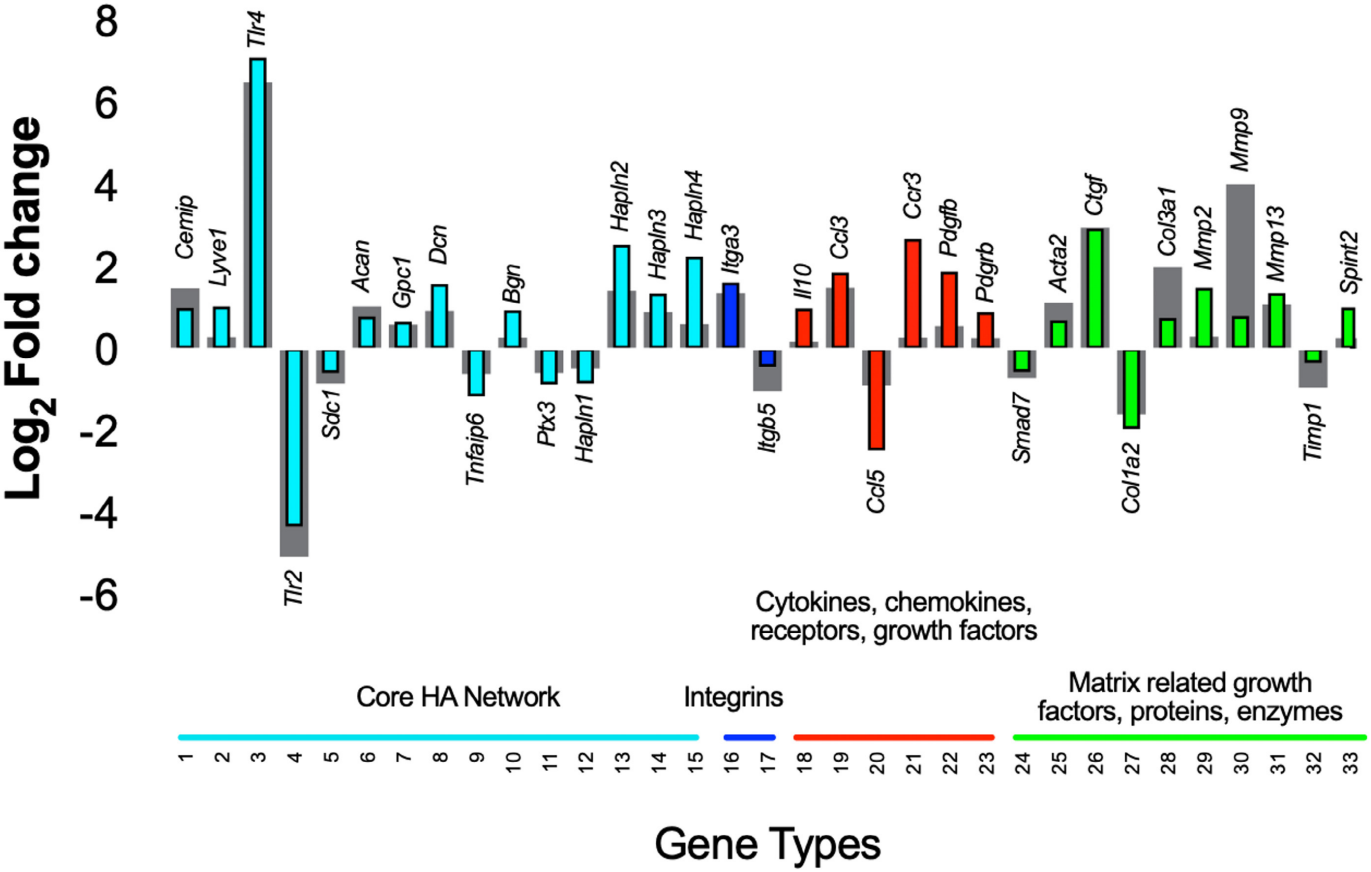
COCs from reproductively old mice demonstrate differential expression of genes broadly involved in HA biology within fibroinflammatory microenvironments compared to reproductively young counterparts. Gene expression fold changes for all 88 genes (see Figure S8 for full list) were log2 transformed to show expression changes relative to zero and plotted by seven categories. This assay was performed twice with the analysis of pooled COCs at 4 h post‐IVM induction from 5 reproductively young (78 COCs for the first and 94 COCs for the second experiment) and 5 old (28 COCs for the first and 36 COCs for the second experiment) mice per experiment. Colored and gray columns display assay 1 and 2 results, respectively. Transcripts that showed a differential expression in the same direction in both experiments and demonstrated an average fold change (FC) of >+1.5 (equivalent to >0.585 on the graphs) for up‐regulation or a FC >−1.5 (equivalent to <−0.599 on the graphs) for down‐regulation are presented here. HA, hyaluronan; IVM, *in vitro* maturation.

To further understand how the HA network may be altered with age in the COC, we developed a Customized RT^2^ Profiler PCR HA Network Array which enabled simultaneous interrogation of the expression of 88 genes broadly involved in HA biology within fibroinflammatory microenvironments (Figure S8). We evaluated COCs at 4 h post‐IVM induction as this is a timepoint when the expression of many genes involved in the cumulus expansion is up‐regulated (Adriaenssens et al., 2011; Dunning et al., 2015; Hernandez‐Gonzalez et al., 2006). Age was associated with differential expression of 33 genes, with 23 genes up‐regulated in COCs from reproductively old mice relative to controls and 10 genes down‐regulated (Figure 4). Interestingly, the expression of *Cemip*, a hyaluronidase, was up‐regulated (~2.4‐fold) with age which is consistent with the overall decrease in HA observed in COCs from reproductively old mice. Moreover, *Sdc1*, *Ptx3*, and *Tnfaip6*, which encode key components of the COC HA matrix (Fülöp et al., 2003; Nagyova, 2018; Salustri et al., 2004, 2019), were down‐regulated (~1.6‐fold, 1.6‐fold, and 1.8‐fold, respectively) in COCs from reproductively old mice. *Hapln1* is a member of HA and proteoglycan binding link protein (*Hapln*) gene family and enhances COC expansion through stabilization of the COC matrix via crosslinking of the HA molecules (Liu et al., 2010). *Hapln1* levels were decreased (~1.5‐fold), whereas *Hapln2*, *Hapln3*, and *Hapln4* levels were increased (~4‐fold, 2‐fold, and 3‐fold, respectively) in COCs of reproductively old mice.

Loss of HA homeostasis is associated with inflammation because low‐molecular mass HA fragments can bind to receptors such as CD44 and Toll‐like receptors (*Tlr*) and stimulate inflammatory signaling cascades (Chang et al., 2007; Jiang et al., 2005; Liu et al., 2008; Shimada et al., 2008). Members of the *Tlr* family are part of the innate immune system and are expressed by cumulus cells during COC expansion and lead to the induction of inflammatory genes (Liu et al., 2008; Turathum et al., 2021). *Tlr4* was up‐regulated (~115‐fold) and *Tlr2* was down‐regulated (~25 fold) in COCs with age. Furthermore, the expression of inflammatory mediators, such as chemokine ligand 3 (*Ccl3*) and chemokine receptor 3 (*Ccr3*), was up‐regulated (~3‐fold and 4‐fold, respectively) in COCs of reproductively old mice. Inflammation is associated with fibrosis, and we noted an age‐dependent increase in expression of platelet derived growth factor B (*Pdgfb*), platelet derived growth factor receptor β (*Pdgfrb*) α smooth muscle actin (*Acta2*), connective tissue growth factor (*Ctgf*), and collagen III (*Col3a1*) (~2.6‐fold, 1.5‐fold, 1.9‐fold, 7.8‐fold, and 2.8‐fold, respectively), suggesting increased fibroblast activation and matrix synthesis, consistent with age‐associated ovarian fibrosis (Amargant et al., 2020).

In addition to fibroinflammatory genes, the expression of matrix‐related genes was altered in COCs with age. The balance between proteolytic activity of matrix metalloproteinases (*Mmps*) and their tissue inhibitors (*Timps*) play an important role in ECM remodeling throughout oocyte maturation, follicle development, and ovulation (Luddi et al., 2018; Smith et al., 2002). *Mmp2*, *Mmp9*, and *Mmp13* levels were increased (~2‐fold, 9‐fold, and 2‐fold, respectively), and *Timp1* levels were decreased (~1.5‐fold) in COCs of reproductively old mice. Overall, essential COC matrix components exhibited reduced expression, and there was a dysregulation in expression of matrix regulators with a shift toward increased ECM degradation. Interestingly, nine of the genes interrogated in COCs exhibited overall similar age‐dependent patterns of gene expression in the whole ovary and ovarian stroma, suggesting general dysregulation of these genes with age within ovarian compartments (Figure S9).

### 3.5 The integrity of the ECM in expanded COCs is compromised with age

**FIGURE 5:**
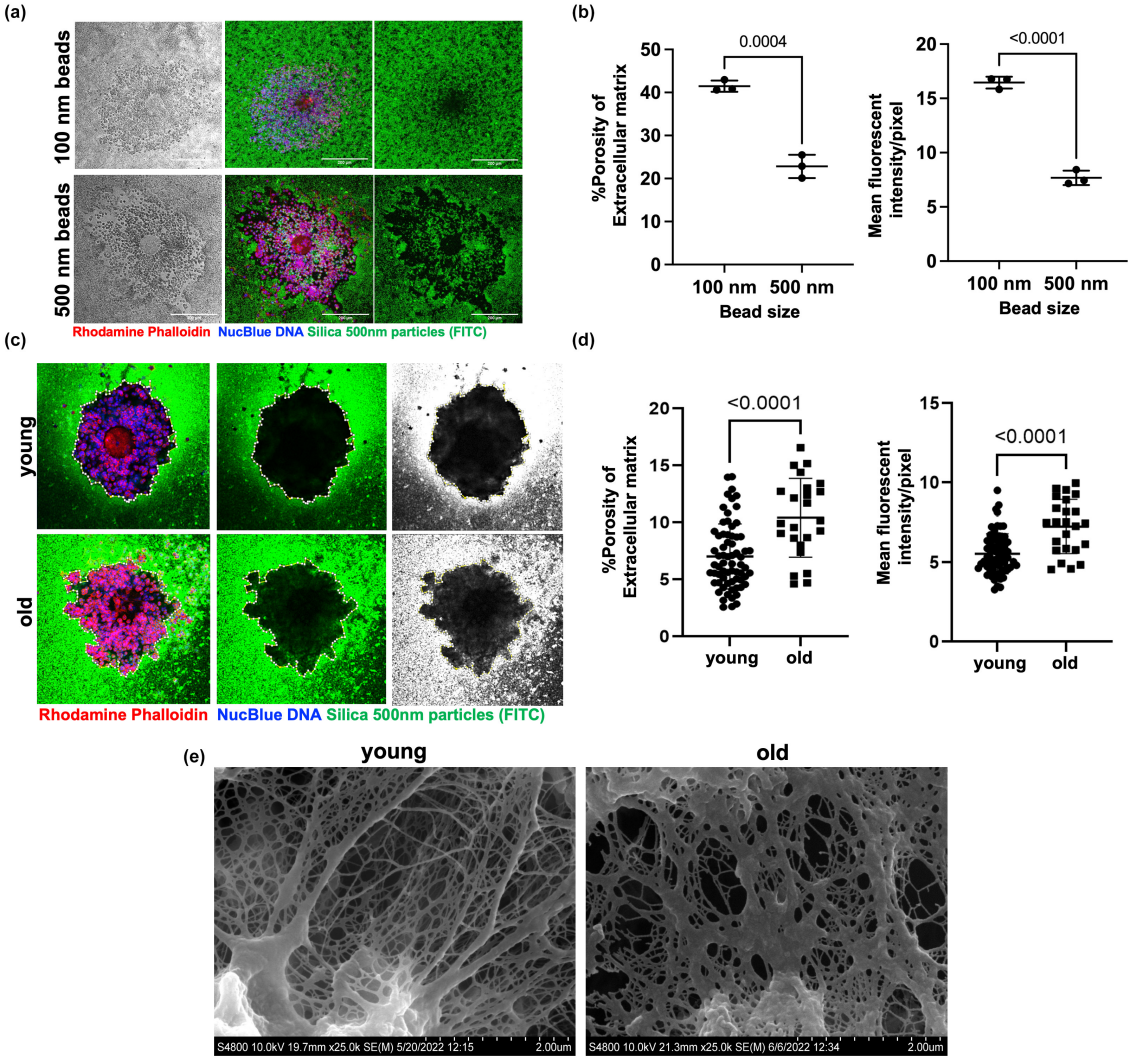
Extracellular matrix (ECM) integrity is compromised with age in expanded COCs. (a) Particle exclusion assay (PEA) was performed on expanded COCs using fluorescent silica nanoparticles of different sizes: Brightfield image (left panel); merge channel (middle panel); green channel (right panel). COCs incubated with 500‐nm nanoparticles demonstrated a clear zone of exclusion at the periphery (scale bars—200 μm). (b) ECM percent porosity (calculated by dividing the area infiltrated by nanoparticles within the COC to the whole area occupied by the COC) and nanoparticle mean fluorescent intensity/pixel of COC image (calculated as the total nanoparticle fluorescent intensity per COC/total number of pixels per COC image) provide quantitative assessment of ECM permeability to these nanoparticles. (c) Representative images of the PEA performed on COCs from reproductively young and old mice using 500‐nm nanoparticles: Merge channel (left panel); green channel (middle panel); grayscale image of the green fluorescent nanoparticles (right panel). The contrast was adjusted similarly across images to highlight particle distribution. (d) Porosity of ECM and nanoparticle mean fluorescent intensity/pixel of COC image demonstrated increased permeability to 500‐nm nanoparticles in expanded COCs of reproductively old mice (young: pooled analysis of 69 COCs from 6 mice, and old: pooled analysis of 24 COCs from 6 mice) (DNA, blue; Actin, red; silica nanoparticles, green). (e) Representative scanning electron microscopy images of expanded COCs from reproductively young and old mice highlighting the matrix region (33 COCs from 5 young and 19 COCs from 5 old mice were used, scale bars 2 μm). Data are represented as mean ± SD. Two‐sided Student's *t*‐test was used for statistical analysis. *p* < 0.05 was considered statistically significant.

To determine whether defective cumulus expansion, reduced HA, and altered expression of HA‐related transcripts have consequences for the integrity of the ECM of the COC with age, we performed a fluorescence‐based particle exclusion assay (PEA; Wei et al., 2019). This assay is used to visualize pericellular HA glycocalyces which form a barrier to particle penetration. Whereas 100‐nm nanoparticles were able to fully penetrate the cumulus layers of expanded COCs from reproductively young mice, 500‐nm nanoparticles exhibited clear areas of exclusion which were visible both by transmitted light and fluorescence microscopy (Figure 5a). This was reflected by the quantitative assessment of matrix integrity with the calculation of matrix porosity, calculated by dividing the area infiltrated by nanoparticles within COC to the whole area occupied by the COC (41.47 ± 1.30% vs. 22.82 ± 2.70%, *p* < 0.001, for 100‐nm and 500‐nm particles, respectively) and the nanoparticle mean fluorescent intensity/pixel of COC image (16.46 ± 0.54 vs. 7.676 ± 0.66, *p* < 0.0001, for 100‐nm and 500‐nm particles, respectively) which demonstrated that the COC ECM was more permeable to 100‐nm particles compared to 500 nm (Figure 5b). This is consistent with a previous report which, based on COC stiffness measurements, estimated that COCs demonstrate high porosity at baseline with the effective mesh size on the order of a few hundred nanometers (Chen et al., 2016). Given the high baseline porosity and high permeability to 100‐nm particles, we decided to use 500 ‐nm particles to evaluate differences in ECM porosity, and thereby integrity, between expanded COCs from reproductively young and old mice. The porosity of the COC ECM (10.42 ± 3.46% vs. 7.02 ± 2.85%, *p* < 0.0001) and nanoparticle mean fluorescent intensity/pixel of COC image (7.25 ± 1.70 vs. 5.55 ± 1.26, *p* < 0.0001) were significantly higher in reproductively old mice compared to young controls (Figure 5c,d). This increased penetration of 500‐nm fluorescent silica nanoparticles demonstrates compromised ECM integrity in expanded COCs of reproductively old mice. To further validate these findings, we performed scanning electron microscopy (SEM) of expanded COCs from reproductively young and old mice to visualize the structure of the matrix. SEM demonstrated increased spaces between the extracellular network of fibers and strands in expanded COCs from reproductively old mice compared to young (Figure 5e). These larger spaces are consistent with the increased fluorescence signal observed in the PEA in expanded COCs with age (Figure 5c,d).

### 3.6 Age‐associated alterations in HA levels in the gamete microenvironment are conserved in humans

**FIGURE 6:**
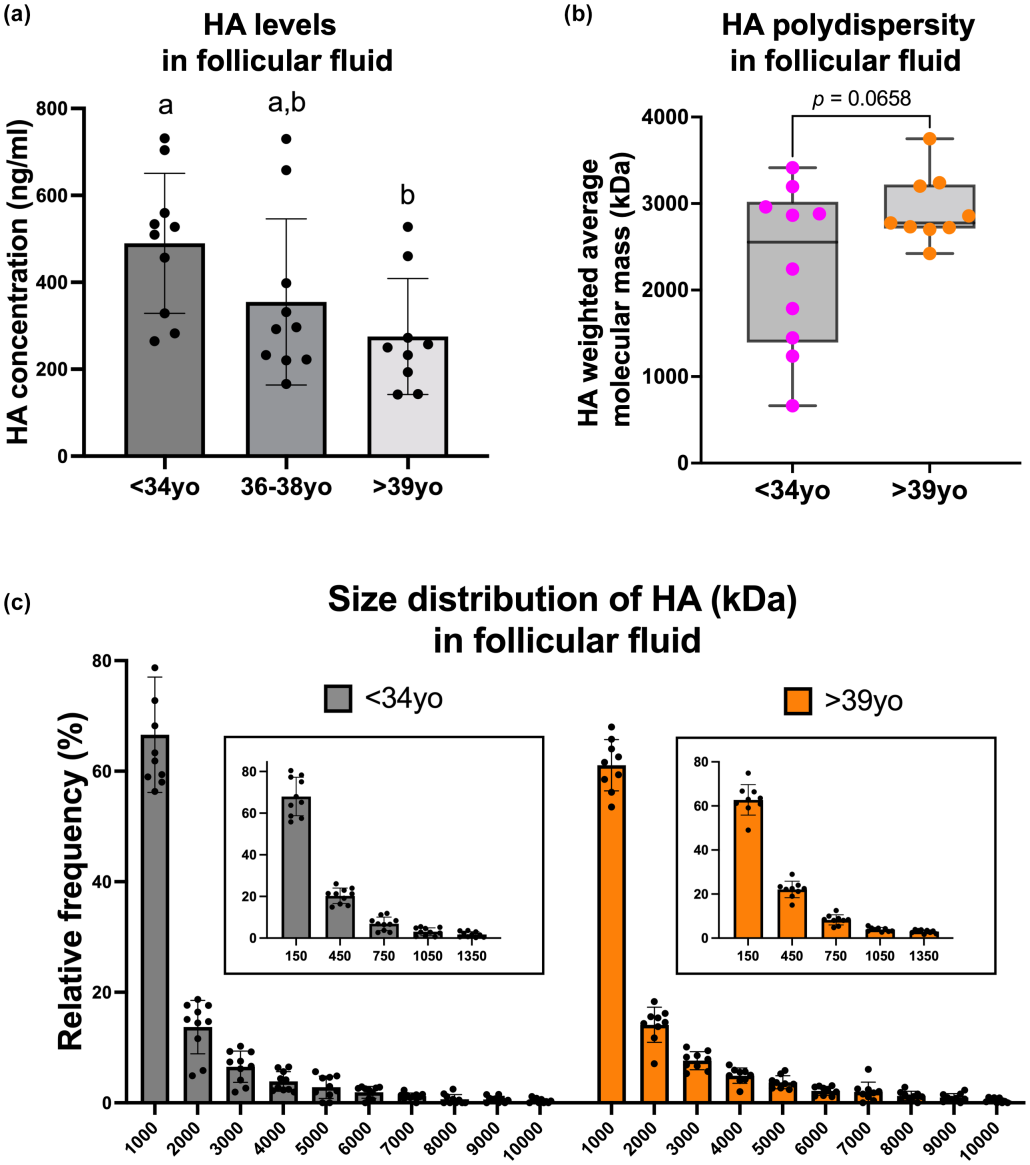
Hyaluronan (HA) levels are decreased in follicular fluid of reproductively older women, and HA polydispersity demonstrates enrichment of low‐molecular mass HA irrespective of age. (a) HA levels demonstrated an age‐dependent decrease in follicular fluid of women undergoing infertility treatment (*n* = 10 for <34 years, *n* = 10 for 36–38 years, *n* = 9 for >39 years group). (b) The weighted average molecular mass of HA displayed a trend toward lower size in follicular fluid of reproductively young women (*n* = 10 for <34 years and *n* = 9 for >39 years group). The box extends from the 25th to 75th percentiles, and the whiskers go from the minimum to the maximum value. (c) HA polydispersity analysis plotted as a frequency distribution histogram demonstrated the enrichment of low‐molecular mass HA in both age groups (*n* = 10 for <34 years and *n* = 9 for >39 years group). Data are represented as mean ± SD. One‐way ANOVA test followed by Tukey's multiple‐comparison tests was used to compare HA levels across age groups. Column bars with different letterheads are significantly different from each other. Two‐sided Student's *t*‐test was used to compare weighted average molecular mass. *p* < 0.05 was considered statistically significant.

Our findings in mice indicated that cumulus expansion was compromised with age due to reduced HA levels and altered expression of HA‐related genes, ultimately resulting in a matrix with compromised integrity. To determine whether similar mechanisms may be conserved in humans, we probed age‐related changes in HA content in follicular fluid which is a gamete microenvironment more readily assayed than human COCs. Follicular fluid is discarded following the removal of the COC for in vitro fertilization (IVF) during routine Assisted Reproductive Technologies. However, this fluid represents the immediate oocyte microenvironment, undergoes changes with reproductive aging, and is rich in HA which is synthesized at the time of ovulation similar to mouse (Babayev & Duncan, 2022; Machlin et al., 2021; Salustri et al., 1992). Therefore, we analyzed the HA content in follicular fluid samples from women in three age groups: <34 years, 36–38 years, and >39 years. Age was the main differentiator across these groups as other clinical parameters were not significantly different (Table S1). HA levels demonstrated an age‐dependent decrease in the follicular fluid of these women (490 ± 161, 355 ± 191 and 275 ± 134 ng/mL in <34 years, 36–38 years, and >39 years group, respectively, *p* < 0.05) (Figure 6a, Figure S10a). Using solid‐state nanopore technology (Rivas et al., 2018, 2022), we analyzed HA size distribution (i.e., polydispersity) in this biological fluid and found that most HA was of low‐molecular mass in both age groups, with HA molecules smaller than 300 kDa being the predominant size (62.7 ± 11.9% and 55.9 ± 7.8%, <34 years, and >39 years, respectively, *p* = 0.16, Figure 6c, Figure S11a). However, HA polydispersity was not significantly different between age groups (Figure 6b, Figures S10b and S12). Nanopore technology cannot reliably identify HA molecules <50 kDa in mass. Therefore, we validated our results via measurement of HA <50 kDa in mass using an HA ELISA‐like assay following molecular weight fractionation of follicular fluid samples. Consistent with what we observed using solid‐state nanopore technology, the levels of <50 kDa HA in follicular fluid of women <34 years were not significantly different relative to the >39 years group (Figure S11b). These data demonstrate that decreasing HA levels in the oocyte microenvironment with advanced reproductive age are conserved in humans.

## 4 DISCUSSION

Reproductive aging and the associated increase in infertility, miscarriage rates, and menopause‐related health sequelae are some of the biggest challenges for healthy aging in women especially as medical interventions continue to extend lifespan. In this study, we demonstrated that impaired and altered morphokinetics of cumulus cell expansion are a feature of advanced reproductive age in mice, and this may contribute to ovulatory dysfunction in vivo given that optimal COC expansion is important for ovulation (Davis et al., 1999; Hizaki et al., 1999; Sato et al., 2001; Varani et al., 2002; Zhuo et al., 2001). Reduced HA levels combined with decreased expression of matrix‐forming genes and increased expression of matrix‐degrading genes underlie cumulus cell matrix dysregulation and loss of integrity. We also demonstrated an age‐associated decrease in human follicular fluid HA levels with advanced age indicating that similar mechanisms are occurring in humans.

It is possible that some of the observed age‐dependent differences in cumulus expansion stem from the observation that the COCs from older animals have fewer cumulus cell layers at baseline. However, the significant decrease in COC area and thickness change (post‐expansion measurements – pre‐expansion measurements), decreased cellular and plasma membrane‐associated HA levels, and altered COC gene expression patterns argue against this as the only mechanism of impaired cumulus expansion with advanced age and highlight the role of HA, HA‐associated ECM components, and their regulation in this process. Other mechanisms involving age‐related dysregulation of LH and EGF‐like growth factor signaling may also contribute to this phenotype given their important roles in cumulus expansion and ovulation (Hsieh et al., 2007; Liu et al., 2014; Park et al., 2004).

Our data demonstrate that even at a young age, oocyte maturation and egg quality can be compromised in a subset of gametes, and it is possible that some of the mechanisms may overlap with those that occur with age. Nevertheless, in this study, we examine cumulus expansion as one such mechanism and show that there are significant age‐dependent differences in this process. This is consistent with broader changes observed with age in cumulus cell function (Babayev & Duncan, 2022). It is also important to note that a disruption of cumulus cell communication with oocytes could be occurring in addition to a defect in cumulus expansion, and in fact, there are decreased transzonal projections between the somatic cells and oocyte with advanced reproductive age (El‐Hayek et al., 2018).

HA production in follicles generates an osmotic gradient and draws water into the antral cavity (Chen et al., 1993). HA is synthesized as high molecular mass (HMM) HA by the activity of HA synthases and is fragmented to low‐molecular mass (LMM) HA by the activity of hyaluronidases or reactive oxygen species (Rowley, Amargant, et al., 2020). While HMM‐HA promotes tissue hydration and homeostasis, LMM‐HA (<250–300 kDa) appears to be proinflammatory (Garantziotis, 2022; Rowley, Amargant, et al., 2020; Snetkov et al., 2020; Tavianatou et al., 2019; Weigel & Baggenstoss, 2017). We have previously demonstrated that LMM‐HA (200 kDa) induces inflammation in ovarian stromal cells, and reduces oocyte maturation rates and leads to abnormal gamete morphology when follicles are grown ex vivo in the presence of these fragments (Rowley, Amargant, et al., 2020; Rowley, Rubenstein, et al., 2020). Whether LMM‐HA also directly impacts inflammatory patterns of gene expression in cumulus cells warrants further investigation.

The decrease in follicular fluid HA levels in humans may contribute to reduced osmotic gradient and smaller dominant antral follicle size with advanced age (Wang et al., 2020). Given this age‐related decrease in follicular fluid HA levels, along with a trend of altered polydispersity, it is tempting to speculate that HA may serve as a non‐invasive extracellular biomarker of oocyte quality. Prospective studies with large patient population are needed to confirm our findings. Future studies will also explore the therapeutic potential of HMM‐HA to rescue the phenotype observed with advanced reproductive age. An in vivo approach will likely be most effective, since HA added to COC culture is unlikely to be incorporated into the COC matrix, and its size and stability cannot be controlled.

Although we are drawing parallels between mice and human regarding their immediate oocyte microenvironment and speculating that reduced HA levels in both cumulus cells and follicular fluid reflect conserved ECM regulation mechanisms, it is worth noting that follicular fluid HA levels may not necessarily reflect those in the cumulus cells. HA in follicular fluid is secreted by cumulus and mural granulosa cells (Salustri et al., 1990, 1992). However, if there is a loss of HA in cumulus cells versus impaired synthesis, this can possibly increase follicular fluid HA levels. Future investigations need to specifically assess HA levels in human cumulus cells as a function of age to answer this question.

Increased proinflammatory gene expression, including up‐regulation of *Tlr4*, in COCs from mice of advanced reproductive age indicates that inflammation may be inherent to the follicle in addition to the extrafollicular ovarian microenvironment (Briley et al., 2016; Foley et al., 2021; Lliberos et al., 2021; Zhang et al., 2020). This is consistent with our previous unbiased transcriptomic analysis of individual mouse follicles that demonstrated increased expression of genes related to immune function, including *Tlr4*, and chemokine signaling with advanced reproductive age (Duncan et al., 2017). Up‐regulated TLR4 levels in COCs from reproductively old mice may represent a compensatory mechanism given its role in inducing *Ifnb1* transcription (Kawai & Akira, 2007; Palsson‐McDermott & O'Neill, 2004) which is important in COC expansion and ovulation in mice (Jang et al., 2015). Future research will shed light on the role of inflammatory COC molecular signature in reproductive aging.

In summary, our study provides novel insights into the age‐dependent changes in the immediate oocyte microenvironment and suggests a potential mechanism of impaired ovulation with advanced reproductive age. This work may have implications for current ART practice where oocyte retrieval in all age groups takes place 34–38 h after the ovulation trigger injection depending on the clinic (Shen et al., 2020). This one‐size‐fits‐all approach may not be appropriate for all ages given the differences in cumulus expansion which is a marker of ovulation trigger response. COCs from reproductively older patients may require more time to achieve full expansion potential and optimal oocyte competence. Age‐related dysfunctional COC expansion with abnormal ECM deposition may also have implications beyond ovulation, such as transfer of the COCs in the oviduct and fertilization. Therefore, this work is important for the understanding of reproductive aging, age‐related increase in subfertility, and treatment of infertility.

## AUTHOR CONTRIBUTIONS

E.B., M.T.P., and F.E.D. conceived the original idea and designed the experiments. E.B., C.S., D.G., W.S.P., and F.P. carried out the experiments. E.B., M.E.P., A.R.H., M.T.P., and F.E.D. analyzed and interpreted the data. E.B. and D.G. wrote the manuscript. A.R.H., M.T.P., and F.E.D. provided critical discussion, reviewed, and revised the manuscript. All authors provided final approval of the manuscript prior to submission.

## FUNDING INFORMATION

This study was supported by the Eunice Kennedy Shriver National Institutes of Child Health and Development (R01 HD093726, M.T.P. and F.E.D.; and K12 HD050121, E.B.). A.R.H. received funding from R01GM134226 and P41EB020594. This work was also supported, in part, by the Bill & Melinda Gates Foundation (INV‐003385, F.E.D.). Under the grant conditions of the Foundation, a Creative Commons Attribution 4.0 Generic License has already been assigned to the Author Accepted Manuscript version that might arise from this submission.

## CONFLICT OF INTEREST STATEMENT

The authors have declared that no conflict of interest exists.

## Supporting information


Appendix S1.

Video S1.

## Data Availability

All data generated in this study are available upon request from the corresponding authors.
