# Supplementary material for: Cumulus expansion is impaired with advanced reproductive age due to loss of matrix integrity and reduced hyaluronan
Source: Aging Cell. 2023 Oct 18;22(11):e14004. doi: 10.1111/acel.14004 (PMC10652338; doi:10.1111/acel.14004)
Supplement: Supplementary file 1 — Appendix S1. [file ACEL-22-e14004-s001.pdf]

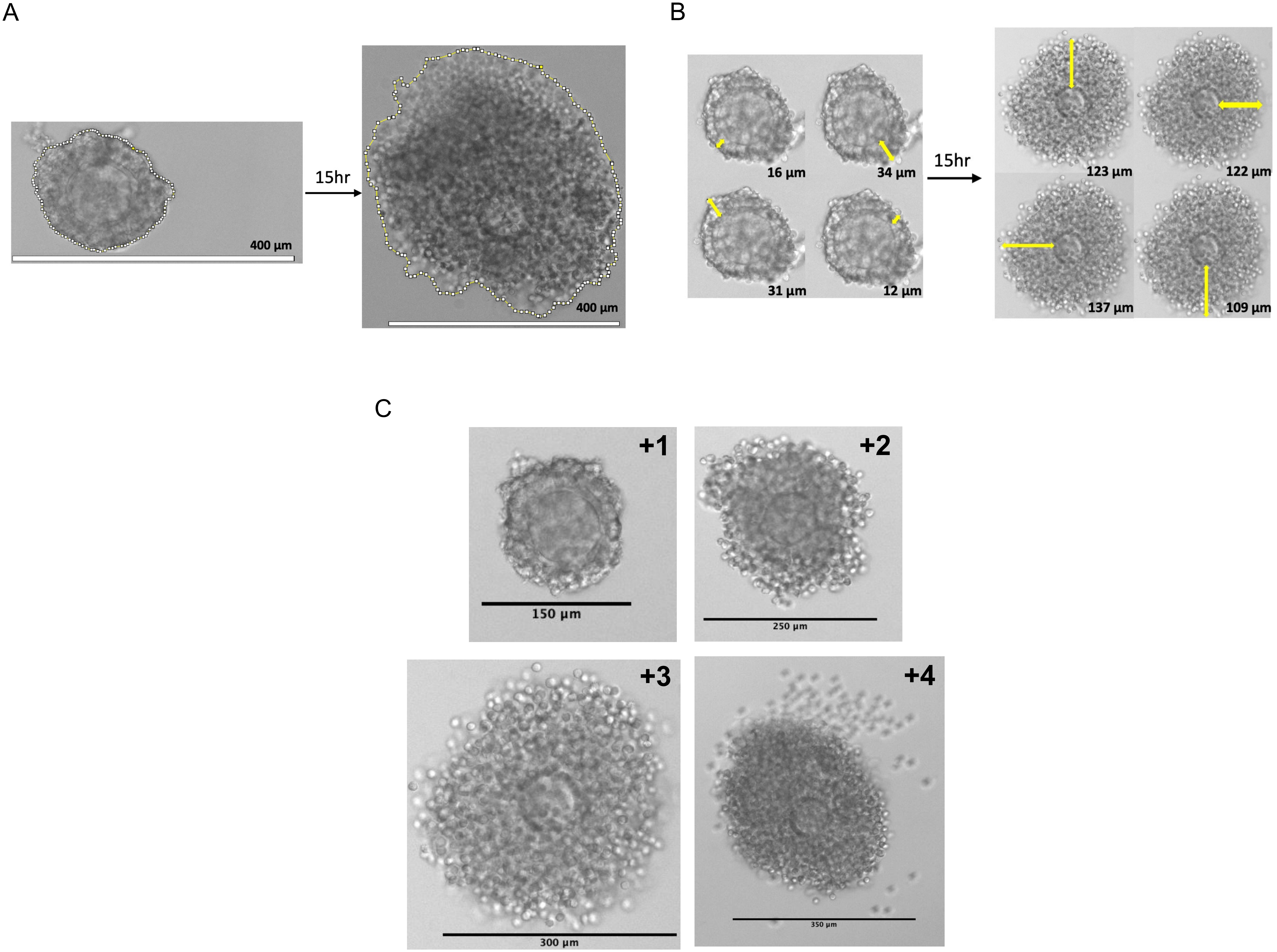

Figure S1. Assessment of cumulus expansion. A) COC area was measured before and after IVM using FIJI (scale bars – 400 mm) B) Cumulus cell layer thickness was measured in 4 quadrants (at 3, 6, 9, 12 o'clock positions) using FIJI and averaged to obtain mean cumulus cell layer thickness. C) Representative images of subjective cumulus expansion scoring. The image brightness/contrast was adjusted for the ease of visualization.

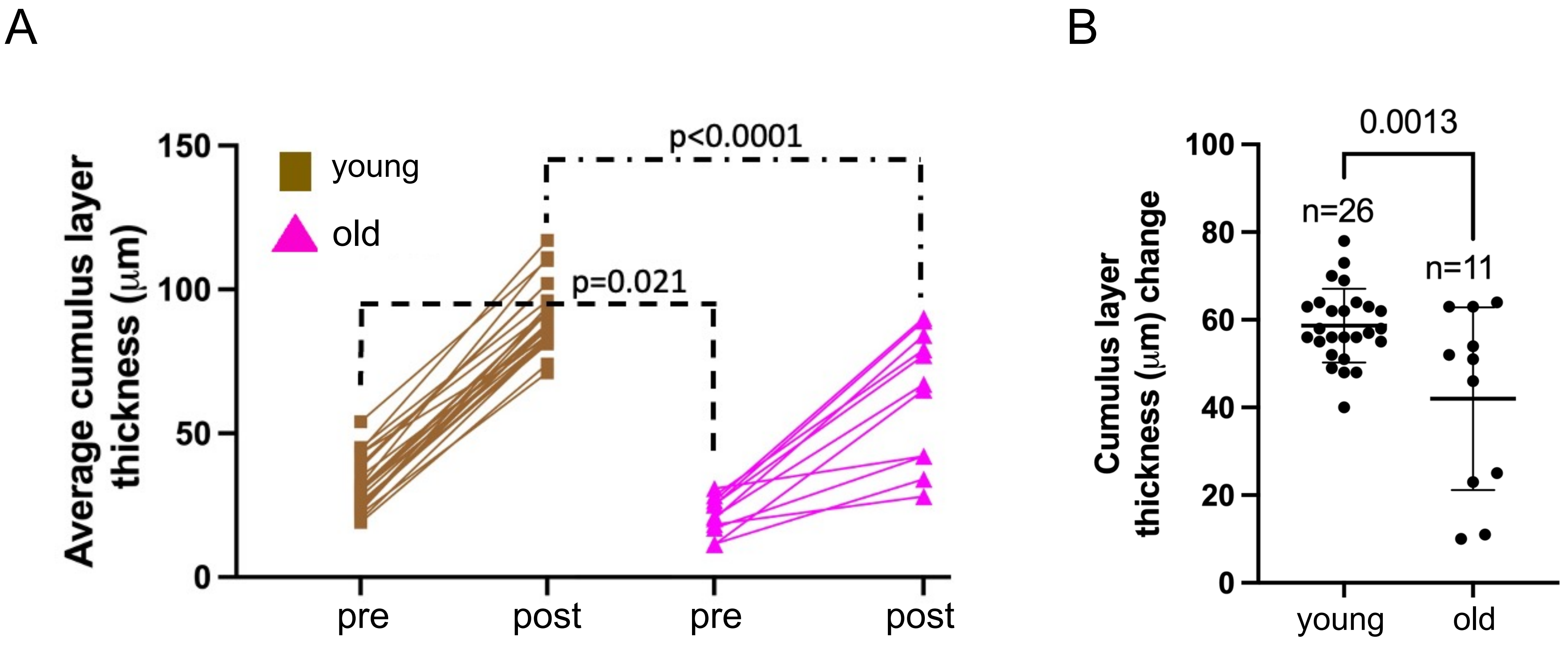

Figure S2. Cumulus expansion is impaired in COCs from reproductively old (14-17 months) CB6F1 mice during IVM. A) Average cumulus cell layer thickness was decreased in COCs from reproductively old mice pre- and post-expansion. B) The change in average cumulus cell layer thickness (post-expansion thickness – pre-expansion thickness) was significantly less in COCs from reproductively old mice COCs during IVM (testing the difference between differences). Data are represented as mean ± SD. Experiments were repeated 5 times with the comparison of all COCs from one young and one old mouse per experiment (5-29 COCs per mouse). Two-sided Student's t-test or Mann-Whitney U test were used to compare continuous variables depending on normality. $P<0.05$ was considered statistically significant.

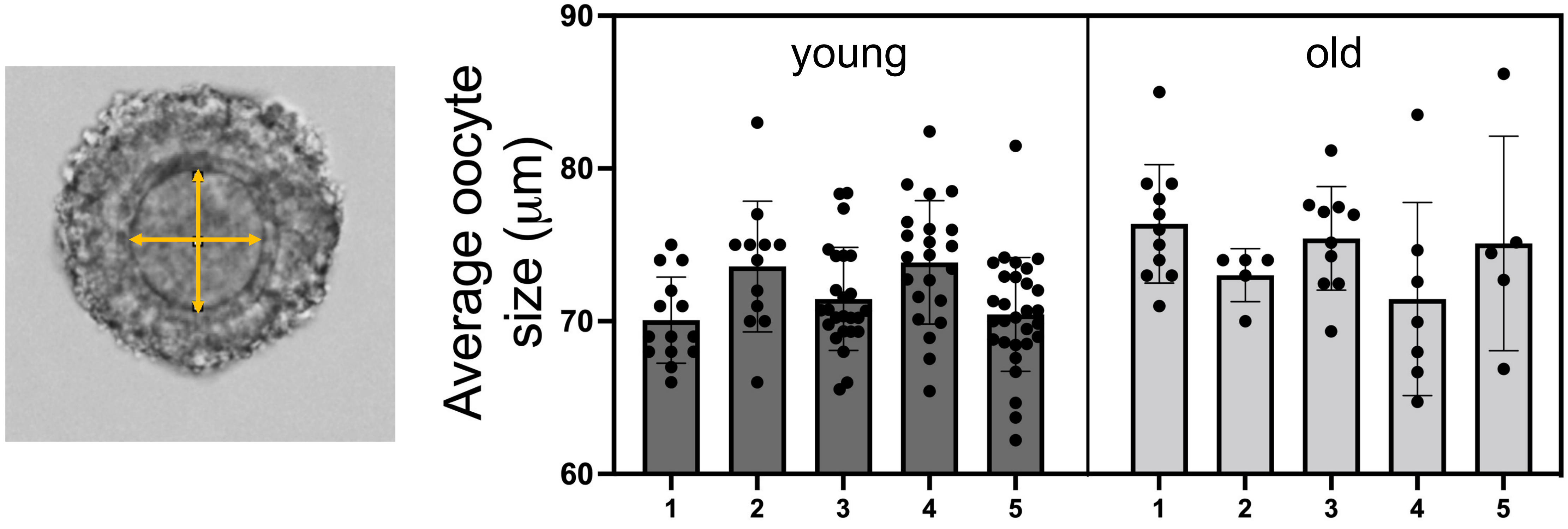

Figure S3. Oocyte size is not different in COCs from reproductively young and old mice. Two perpendicular measurements across oocytes were taken using FIJI and averaged to obtain mean oocyte diameter. Average oocyte size was not different between COCs from reproductively young and old mice (70-74 ± 2.8-4.3 µm versus 71-76 ± 1.7-7.0 µm). Each bar represents data from an individual mouse (n=5 in each group) with individual dots on the scatter plot representing oocyte diameter with mean ± SD displayed. Nested t-test was used for statistical comparison.

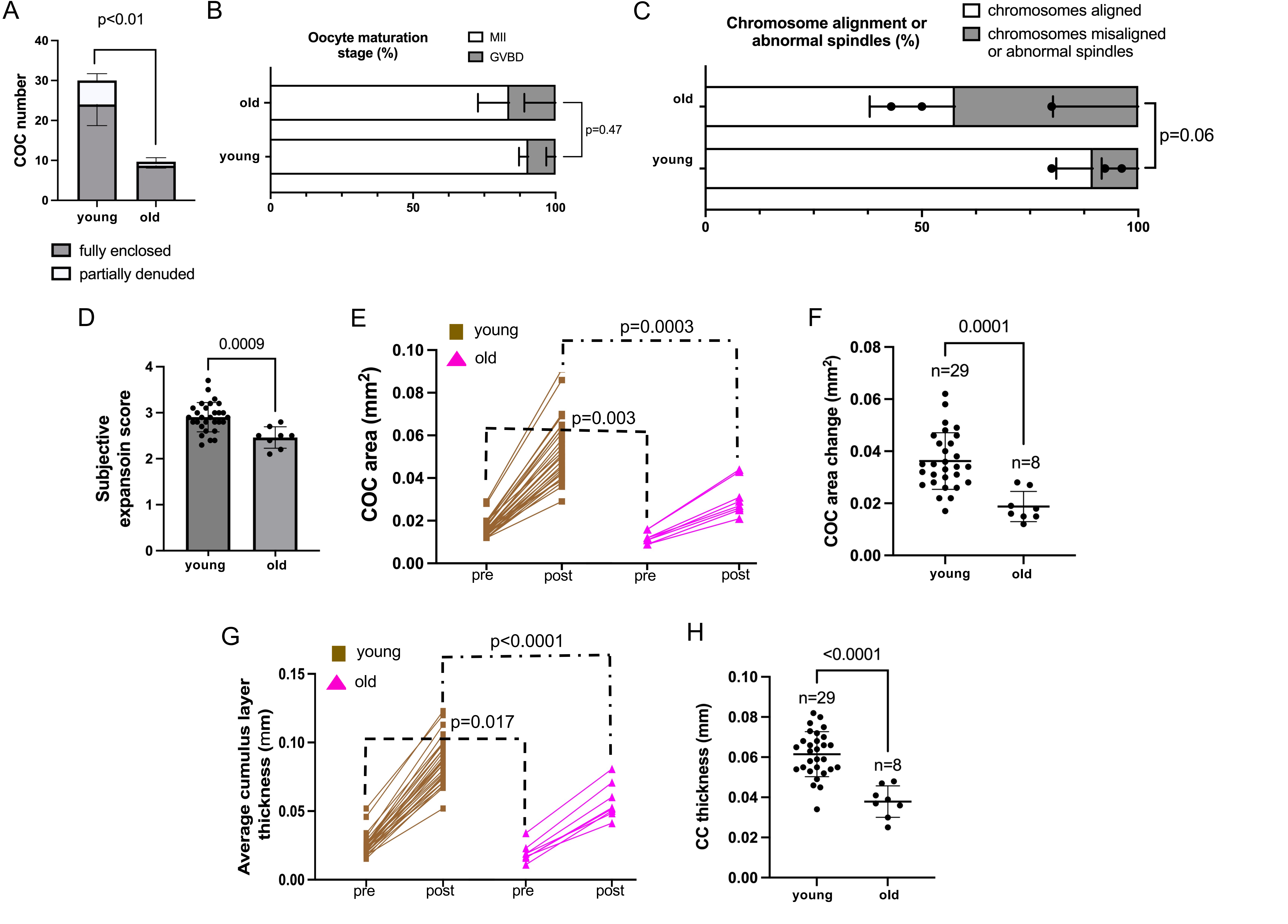

Figure S4. Cumulus expansion is impaired in COCs from reproductively old CD1 mice during IVM A) Significantly fewer COCs were obtained from reproductively old mice. B) Oocyte maturation stages were not different between reproductively young and old mice after IVM. C) Chromosome alignment on metaphase II spindles showed a trend towards increased abnormalities in older mice (black dots represent the proportion of oocytes with normally aligned chromosomes per experiment). D) COCs from reproductively old mice exhibited decreased subjective expansion scores. E) COC area was decreased pre- and post-expansion in COCs from reproductively old mice. F) The change in COC area (post-expansion area – pre-expansion area) was significantly less in COCs from reproductively old mice COCs during IVM (testing the difference between differences). G) Average cumulus cell layer thickness was decreased in COCs from reproductively old mice pre- and post-expansion. H) The change in average cumulus cell layer thickness (post-expansion thickness – pre-expansion thickness) was significantly less in COCs from reproductively old mice COCs during IVM (testing the difference between differences). Data are represented as mean ± SD. Experiments were repeated 3 times with all COCs from 2 young and 2 old mice pooled per experiment (8-29 COCs per group). Two-sided Student's t-test or Mann-Whitney U test were used to compare continuous variables depending on normality. Chi-square test was used to compare categorical variables. P<0.05 was considered statistically significant. GVBD-germinal vesicle breakdown, MII-metaphase of meiosis II

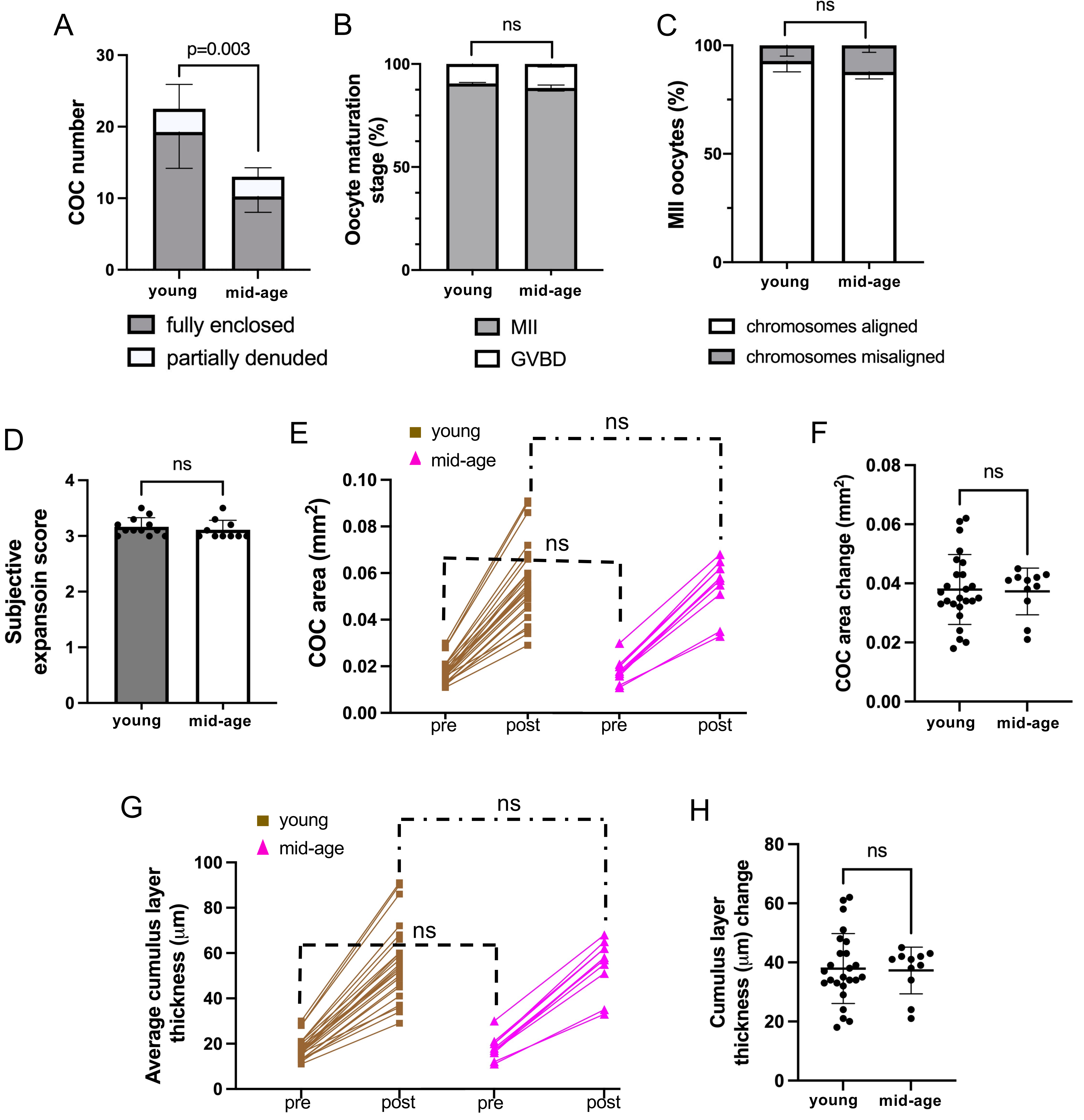

Figure S5. Cumulus expansion is not altered during IVM in COCs isolated from mid-reproductive age CD1 mice. A) Fewer COCs were obtained from mice at mid-reproductive age. B) Oocyte maturation stages, C) Chromosome alignment on metaphase II spindles, D) COC subjective expansion scores, E) COC area pre- and post-expansion, F) The change in COC area, G) Average cumulus cell layer thickness, and H) The change in average cumulus cell layer thickness were not significantly different between reproductively young and mid-age mice after IVM of COCs in a conventional incubator. Data are represented as mean ± SD. Experiments were repeated 3 times with all COCs from 2 young and 2 old mice pooled per experiment (10-25 COCs per group). Two-sided Student's t-test or Mann-Whitney U test were used to compare continuous variables depending on normality. Chi-square test was used to compare categorical variables. P<0.05 was considered statistically significant. GVBD-germinal vesicle breakdown, MII-metaphase of meiosis II

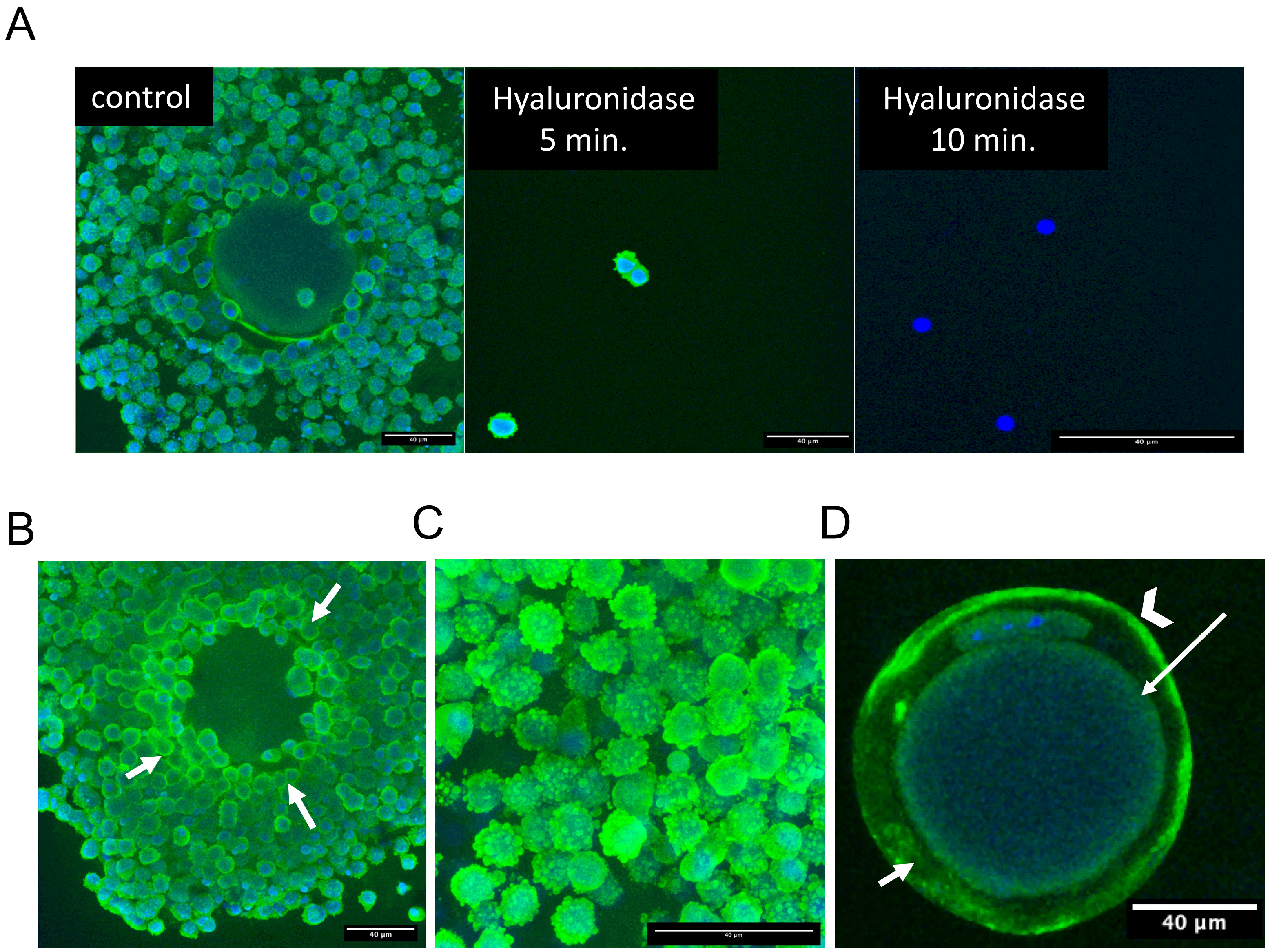

Figure S6. HA staining of expanded mouse COCs using the HABP assay. A) The loss of fluorescent signal after hyaluronidase treatment validated the specificity of the HABP assay. B) HA levels were higher in the corona radiata (i.e., in cumulus cell layer immediately surrounding the oocyte; arrows). C) Plasma membrane blebbing was observed on cumulus cells at high magnification. D) HA staining was detected on the zona pellucida (arrowhead), in the perivitelline space (short arrow) and at the plasma membrane (long arrow). Scale bars – 40 mm.

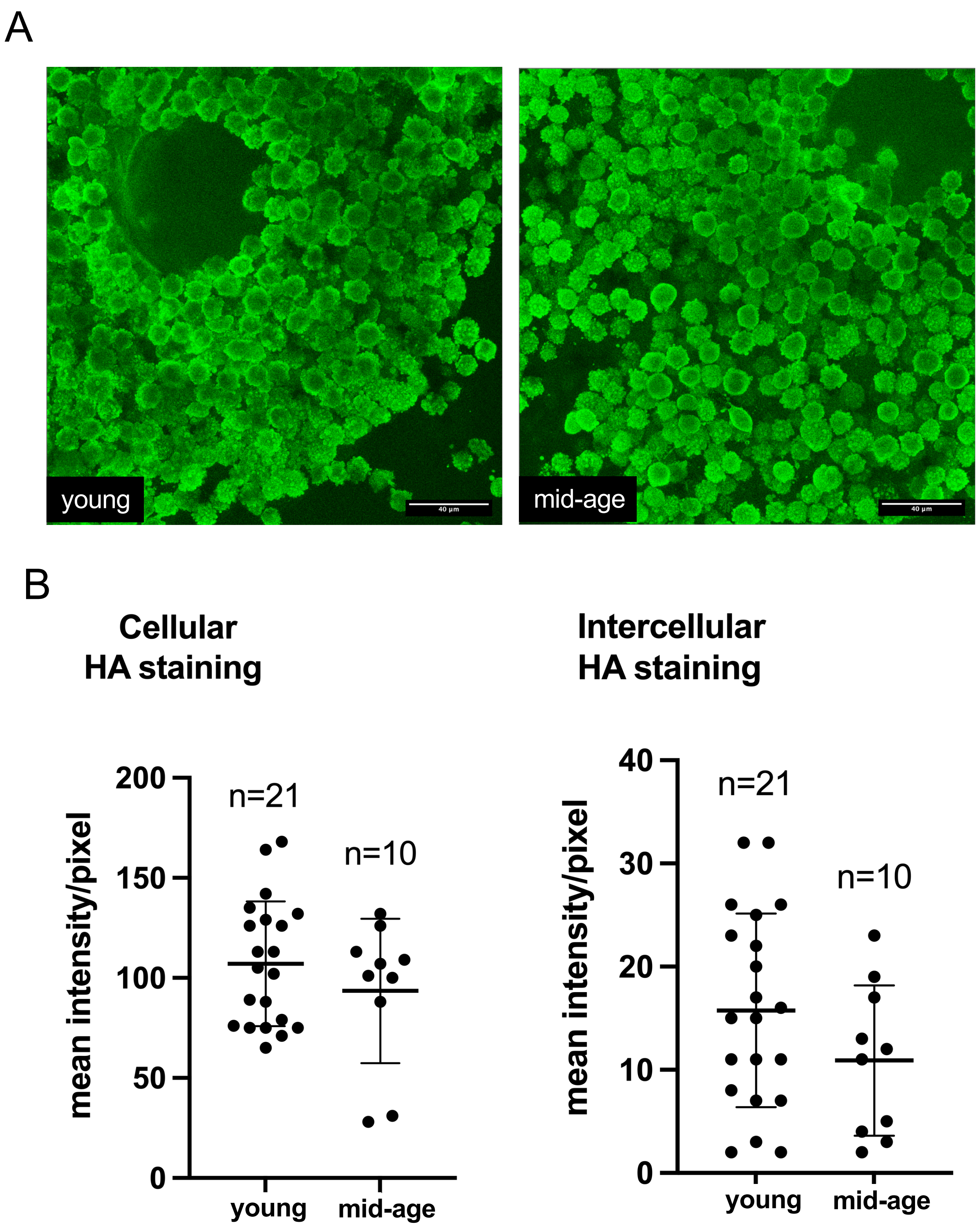

Figure S7. HA levels are not different in expanded COCs from reproductively young and mid-reproductive age CD1 mice. A) Representative images of expanded COCs from reproductively young and mid-age CD1 mice in which HA was visualized using the HABP assay (scale bars – 40 mm). B) Cellular and intercellular HA levels were not altered in expanded COCs of reproductively mid-age CD1 mice. Data are represented as mean ± SD. Experiments were repeated 3 times with the comparison of all COCs from one young and one old mouse per experiment (4-21 COCs per mouse). Two-sided Student's t-test or Mann-Whitney U test were used to compare continuous variables depending on distribution. $P<0.05$ was considered statistically significant.

Color	Category
Cyan	Core HA Network (HA synthesis and degradation enzymes, receptors, binding proteins, and proteoglycans)
Blue	Integrins
Light orange	Vasculature
Orange	Signaling
Magenta	PTM (post-translational modification; O-GlcNAc)
Red	Cytokines, chemokines, receptors and growth factors (non-Tgfb)
Green	General matrix-related (Tgfb/signaling, matrix proteins, crosslinking enzymes, degrading enzymes)
Pink	Housekeeping genes
Olive	Controls (RTC-reverse transcriptase control, PPR-possitive PCR control; GDC-genomic DNA control)

Position	Symbol	Position	Symbol	Position	Symbol	Position	Symbol	Position	Symbol
1	*Has1*	31	*Itga3*	43	*Tnf*	64	*Tgfb1*	89	*Gusb*
2	*Has2*	32	*Itgav*	44	*Il1b*	65	*Tgfb3*	90	*Actb*
3	*Has3*	33	*Itgam*	45	*Il5*	66	*Thbs2*	91	*Gapdh*
4	*Hyal1*	34	*Itgb5*	46	*Il6*	67	*Smad2*	92	*B2m*
5	*Hyal2*	35	*Itgb8*	47	*Il10*	68	*Smad3*	93	*Hsp90ab1*
6	*Tmem2*	36	*Pecam1*	48	*Tnfrsf11a*	69	*Smad4*	94	RTC
7	*Cemip*	37	*Yap1*	49	*Tnfsf11*	70	*Smad7*	95	PPC
8	*Cd44*	38	*Ctnnb1*	50	*Ccl2*	71	*Acta2*	96	GDC
9	*Hmmr*	39	*Wnt5a*	51	*Ccl3*	72	*Ctgf*		
10	*Stab2*	40	*Jun*	52	*Ccl5*	73	*Bambi*		
11	*Lyve1*	41	*Mgea5*	53	*Ccl11*	74	*Grem1*		
12	*Layn*	42	*Ogt*	54	*Ccl12*	75	*Col1a1*		
13	*Tlr4*			55	*Ccr2*	76	*Col1a2*		
14	*Tlr2*			56	*Ccr3*	77	*Col3a1*		
15	*Vcan*			57	*Pdgfb*	78	*Lox*		
16	*Sdc1*			58	*Pdgfra*	79	*Spp1*		
17	*Acan*			59	*Pdgfrb*	80	*Mmp2*		
18	*Gpc1*			60	*Vegfa*	81	*Mmp8*		
19	*Dcn*			61	*Vegfb*	82	*Mmp9*		
20	*Tnfaip6*			62	*Egfr*	83	*Mmp13*		
21	*Bgn*			63	*Adgre1*	84	*Timp1*		
22	*Hspg2*					85	*Serpine1*		
23	*Ptx3*					86	*Plat*		
24	*Hapln1*					87	*Plau*		
25	*Hapln2*					88	*Spint2*		
26	*Hapln3*								
27	*Hapln4*								
28	*Itih2*								
29	*Itih3*								
30	*Itih4*								

Figure S8. List of genes analyzed in the customized RT^2 Profiler PCR Hyaluronan Network array and corresponding category key. The genes were categorized into 7 groups. The fifth column displays 5 housekeeping genes and 3 controls of the array experiment. RTC - reverse transcriptase control; PPC - Positive PCR control; GDC - genomic DNA control.

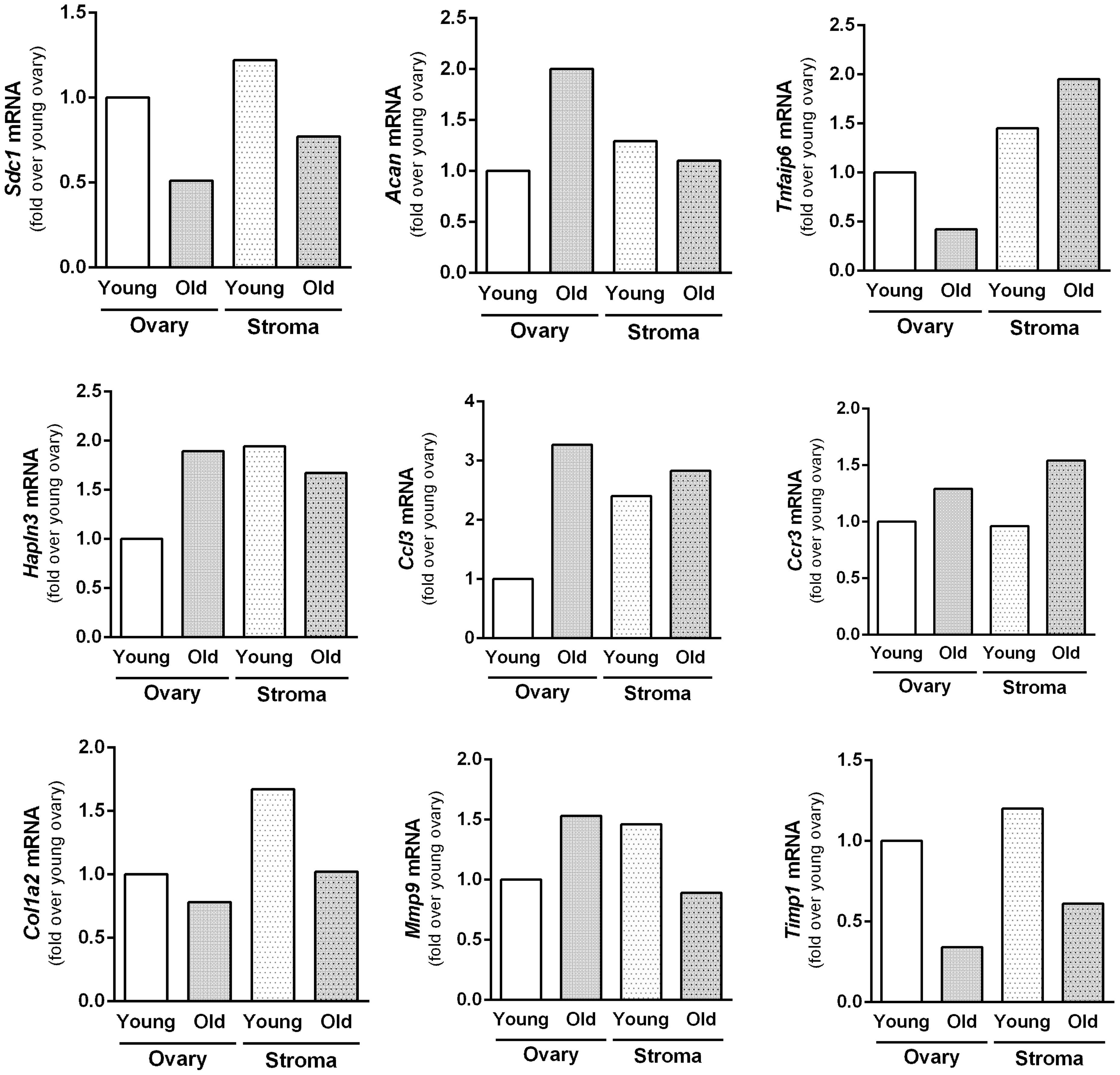

Figure S9. Whole ovaries and enriched stromal fractions from reproductively young and old mice demonstrate similar differential expression of 9 genes analyzed in COCs. Whole ovaries and stromal compartments enriched from pooled ovaries of 4 reproductively young and 4 old mice were used once to perform the same customized RT^2 Profiler PCR Hyaluronan Network array reported in Figure 5. Nine out of 88 transcripts exhibited age-related differential patterns of gene expression that were similar to those observed in COCs.

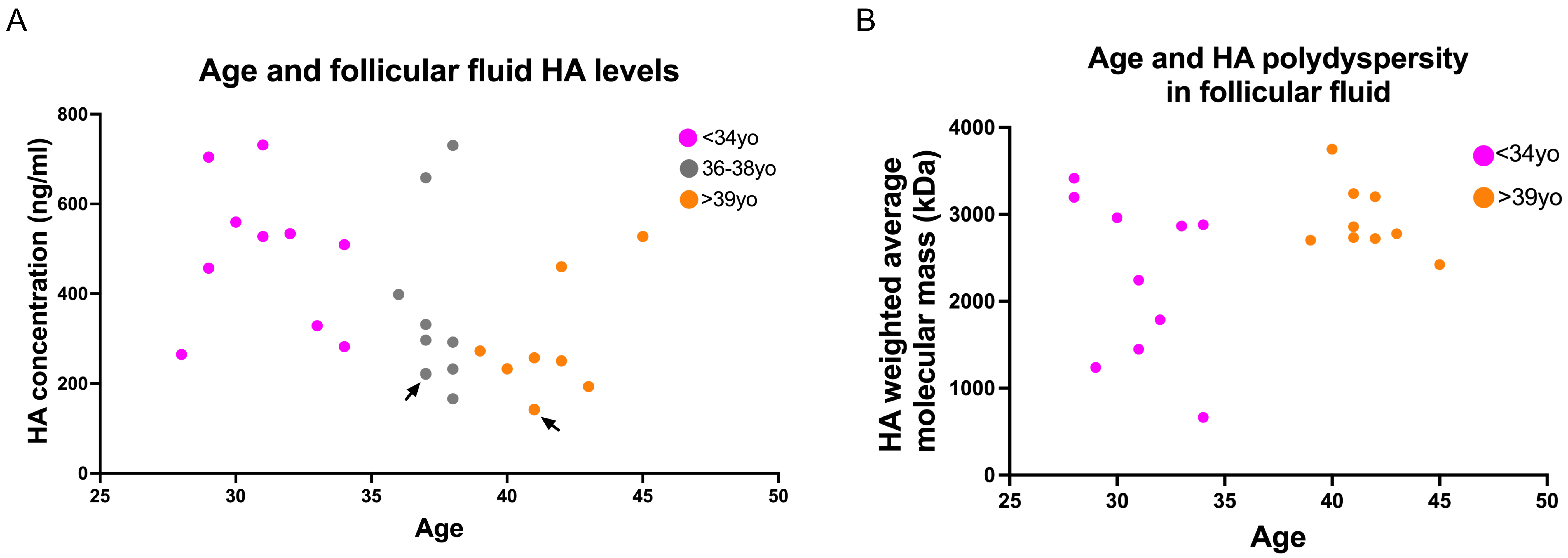

Figure S10. HA levels (A) and weighted average molecular mass of HA (B) in follicular fluid of women undergoing infertility treatment displayed as a scatterplot for each individual patient. Arrows in A point to 2 circles represented by 2 patients each – these patients had the same age and very similar HA levels.

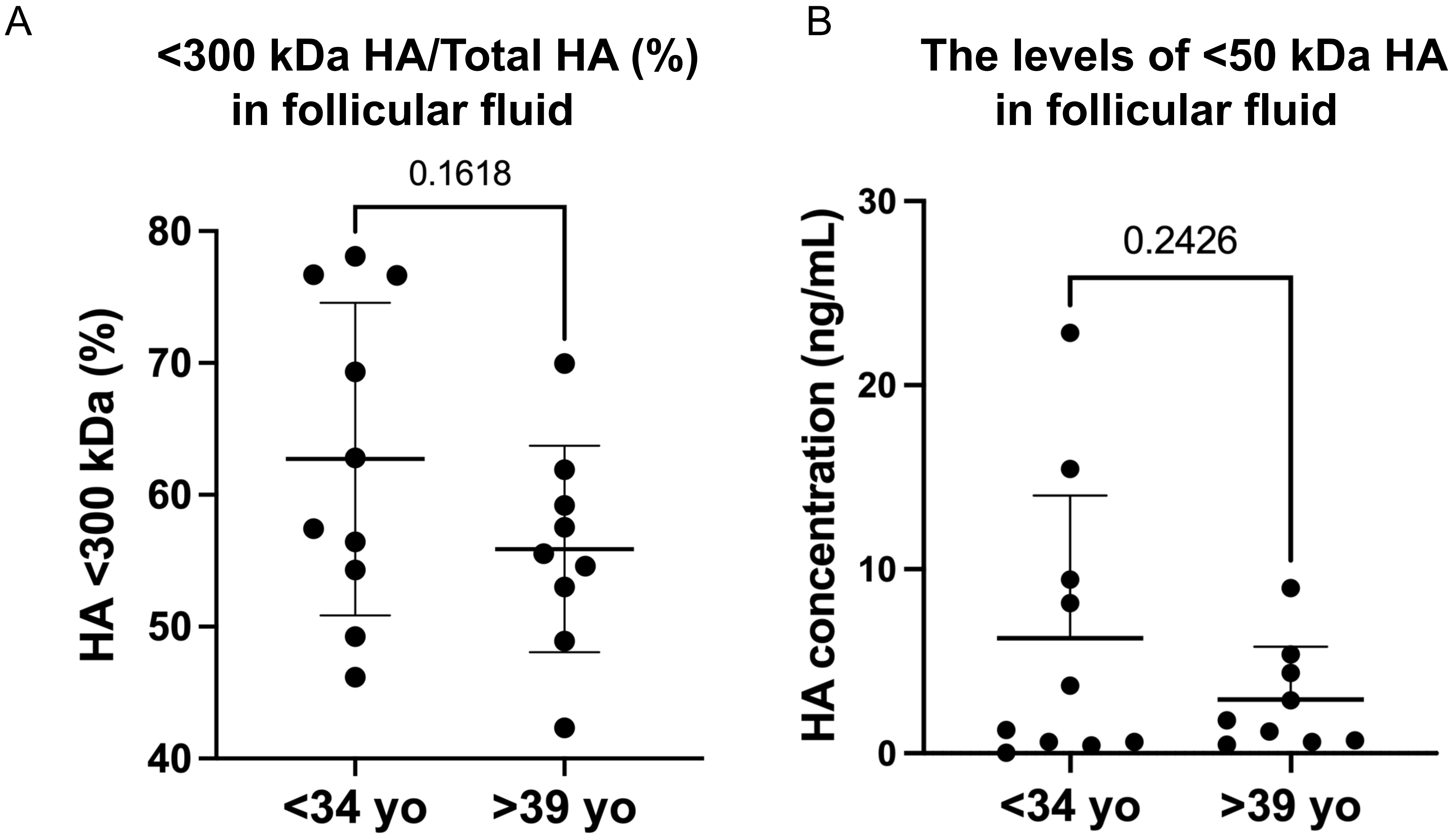

Figure S11. Low-molecular mass HA in follicular fluid of women undergoing infertility treatment. A) <300 kDa was the predominant HA size in follicular fluid of women in <34 yo and >39 yo, with a trend towards higher proportion in follicular fluid of reproductively young women. B) <50 kDa HA levels demonstrated a trend towards higher levels in follicular fluid of reproductively young women (n=10 for <34 yo and n=9 for >39 yo group). Data are represented as mean ± SD. Two-sided Student's t-test or Mann-Whitney U test were used to compare continuous variables depending on distribution. P<0.05 was considered statistically significant.

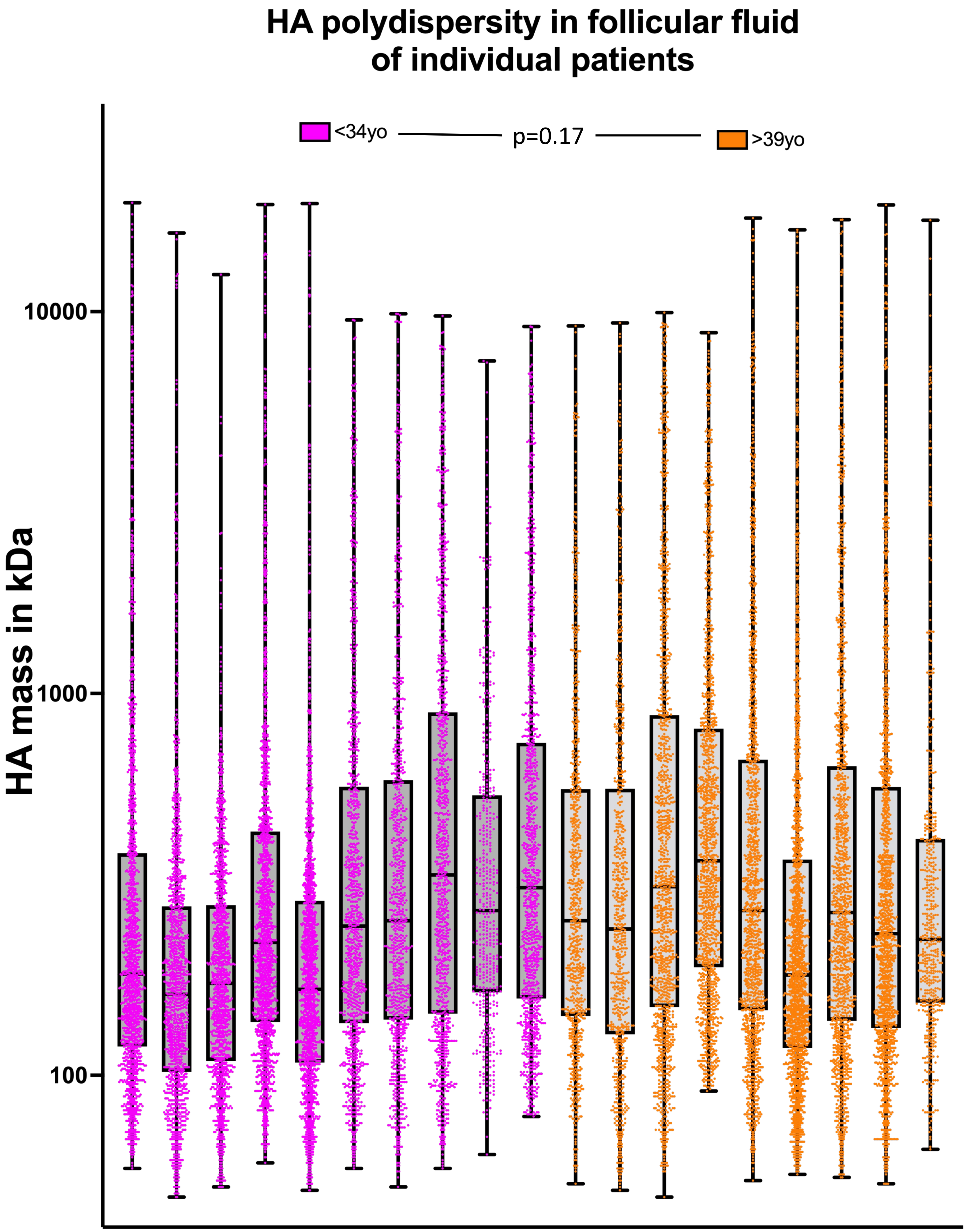

Figure S12. HA polydispersity in follicular fluid of women undergoing infertility treatment is not different between younger and older women (n=10 for <34 yo and n=9 for >39 yo group)**.** Each box and whiskers plot represents an individual patient. Each colored dot represents the size of an individual HA molecule for the patient. The box extends from the 25th to 75th percentiles, and the whiskers go from the minimum to the maximum value. Data were analyzed under a Box-Cox transformation Ynew = (Y^(-.4242) – 1)/(-0.4242) using a nested t-test to account for the correlation between measures on the same patient.

Table S1. Characteristics of infertility patients who underwent follicular fluid hyaluronan level and polydispersity analysis. AMH - Anti-Müllerian hormone, DOR - diminished ovarian reserve, PCOS - Polycystic ovary syndrome

Groups	<34 yo (n=10)	36-38 yo (n=10)	>39 yo (n=10)	P value
**Age**	31.1 ± 2.0	37.2 ± 0.5	41.2 ± 1.6	<0.0001
**BMI (kg/m^2)**	23.2 ± 3.3	24.2 ± 5.3	26.8 ± 4.5	0.2
**AMH (ng/ml)**	3.7 ± 3.7	2.2 ± 1.9	2.0 ± 2.2	0.3
**Days of ovarian stimulation**	10.4 ± 1.4	10.3 ± 1.6	9.4 ± 1.3	0.3
**Peak estradiol level (pg/ml)**	4415 ± 1406	2079 ± 1012	2052 ± 1087	0.2
**Infertility diagnosis**	4 unexplained<br>2 male factor<br>2 tubal factor<br>1 DOR<br>1 PCOS	3 unexplained<br>3 male factor<br>3 DOR<br>1 PCOS	4 unexplained<br>3 DOR<br>3 tubal factor	
